# Who’s there? – First morphological and DNA barcoding catalogue of the shallow Hawai’ian sponge fauna

**DOI:** 10.1371/journal.pone.0189357

**Published:** 2017-12-21

**Authors:** Laura Núñez Pons, Barbara Calcinai, Ruth D. Gates

**Affiliations:** 1 Dept. of Biology and Evolution of Marine Organisms (BEOM) Stazione Zoologica ‘Anton Dohrn’ (SZN), Villa Comunale Naples, Italy; 2 Smithsonian Tropical Research Institute, Balboa, Ancón, Ciudad de Panamá, Panamá; 3 Dipartimento di Scienze della Vita e dell’Ambiente, Università Politecnica delle Marche, Via Brecce Bianche, Ancona, Italy; 4 Hawai'i Institute of Marine Biology, University of Hawai'i at Mänoa, Lilipuna rd. Kane'ohe, HI, Hawai'i, United States of America; University of Guelph, CANADA

## Abstract

The sponge fauna has been largely overlooked in the Archipelago of Hawai’i, notwithstanding the paramount role of this taxon in marine ecosystems. The lack of knowledge about Porifera populations inhabiting the Hawai’ian reefs limits the development of ecological studies aimed at understanding the functioning of these marine systems. Consequently, this project addresses this gap by describing the most representative sponge species in the shallow waters of the enigmatic bay of Kane’ohe Bay, in O’ahu Island. A total of 30 species (28 demosponges and two calcareous sponges) living associated to the reef structures are here reported. Six of these species are new records to the Hawai’ian Porifera catalogue and are suspected to be recent introductions to these islands. Morphological descriptions of the voucher specimens are provided, along with sequencing data of two partitions involving the mitochondrial cytochrome oxidase subunit 1 (COI) marker and a fragment covering partial (18S and 28S) and full (ITS-1, 5.8S and ITS-2) nuclear ribosomal genes. Species delimitations based on genetic distances were calculated to valitate how taxonomic assignments from DNA barcoding aligned with morphological identifications. Of the 60 sequences submitted to GenBank ~88% are the first sequencing records for the corresponding species and genetic marker. This work compiles the first catalogue combining morphological characters with DNA barcoding of Hawai’ian sponges, and contributes to the repository of public databases through the Sponge Barcoding Project initiative.

## Introduction

**Fig 1:**
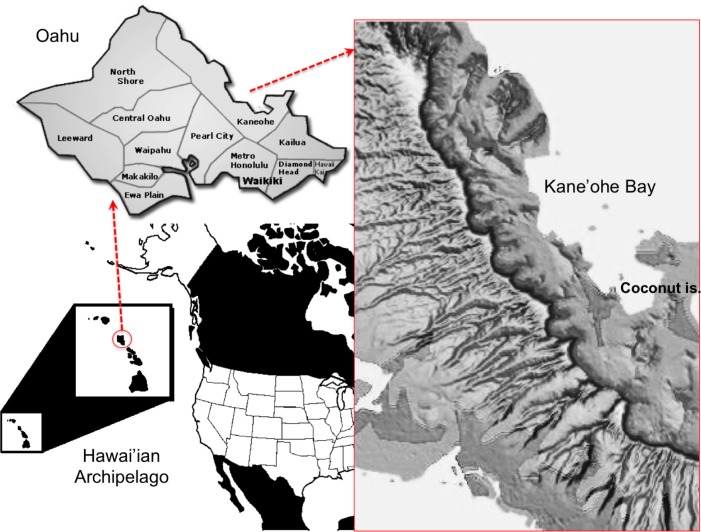
Map of major shipping routes around the Hawai’ian Archipelago. Detail of Kane’ohe Bay in Oahu Island, and the sampling reefs: 20, 22, 25, 44 and HIMB (Hawai’i Institute of Marine Biology). Images were downloaded for illustrative purposes only from public domains (http://claver.gprep.org/sjochs/historical-blank_maps_for_quizzes-.htm, and USGS National Map Viewer at http://viewer.nationalmap.gov/viewer/).

The Hawai’ian Archipelago lies near the centre of the north tropical Pacific Ocean. It is the most isolated land area in the world: >4300 km from North America and the South Pacific continental lands and >6400 km away from Japan. Hawai’i is actually one of the regions with the highest levels of endemism [1]. Prior to the arrival of Europeans in 1778, the biota was mostly autochthonous. At the times, basically the only chance for inoculation of marine benthic organisms was by drifting objects or via Polynesian embarkations (canoes) arriving to the islands. Since the 1840’s the development of Hawai’i as a maritime crossroads increased the occurance of introductions of nonindigenous species (NIS) through Indo-Pacific/Philippines routes, as well as other inter-oceanic passages from the Atlantic/Caribbean through the Panama Canal [2,3]. Kane‘ohe Bay (K-Bay), located in the middle windward side of the midst Hawai’ian island of O’ahu, extends 11 km long and 3 km wide along the coast [4]. This bay was a pristine Hawai’ian settlement until the second half of the 20^th^ century, when the water quality degraded due to shipping, intense urbanization, sewage, aquaculture, and development a the US Marine Corp Base. Benthic communities also started to introduce NIS from the western (17%) and central Indo-Pacific (11%), or the Caribbean (11%), particularly on harbours, piers and docks, mostly towards the southern end of the bay near Coconut Island (Moku O Lo'e, Reef 1, see Fig 1) [5]. K-Bay is unique for being a large semi-restricted area with both, estuarine environments, and scattered fringing and patchy coral reefs forming shallow plateaus. Much of the outer perimeter of these patch reefs is living *Montipora capitata*, *Porites compressa* and *Pocillopora damicornis* scleractinian corals, but inside the living ring is often rubble, which renders settlement for sponge populations. The deeper bottom of the bay (>10 m) is sandy and practically no sponges or corals are found here [4]. Kane’ohe Bay provides a natural laboratory where many international scientists develop their work affiliated with the Hawai’i Institute of Marine Biology (HIMB), sited on Coconut Island. Surprisingly, the shallow-water sponge fauna has been largely overlooked, and the scarce taxonomic descriptions come from surveys from the 50’s and 60’s by de Laubenfels [4,6,7,8] and Bergquist [9,10]. De Laubenfels already emphasized that almost any conceivable kind of sponge could be discovered in upcoming dredge hauls, already reflecting the already high incidence of species inoculation. More recently Kelly-Borges and Valentine [11] provided a list of 99 sponges from Hawai’i, and Calcinai et al. 2013 [12] reported other additional five species. The majority of these species are reported from the Western Pacific and some are considered new introductions [10]. We suspected that the number of species and taxonomic designations from those initial catalogues could have changed since, especially with the advent of molecular tools. Therefore, we proceeded to describe the actual Porifera diversity of this symbolic bay by combining classical taxonomy with DNA barcoding, and by depositing the analysed specimens as vouchers in type collections.

Porifera are notoriously difficult to classify by morphological characters, even by experts, and this has yielded uncertainties and undescribed specimens in many biodiversity surveys [13]. For these reasons molecular tools–e.g., barcoding, the use of species-specific sequences to resolve taxonomy [14]–became very popular around spongeologists [15]. Currently, a 2.5% divergence in the standard partition of the mtDNA cytochrome oxidase subunit 1 (COI) marker is an accepted rule to delimit species for eukaryotic barcoding [14,16]. However, this partition seems to be highly conserved in diploblastic animals like sponges, and does not disclose sufficient variability [17,18]. Several sibling sponge species have demonstrated a lack of discrimination based on the COI classical Folmer fragments, when applying the 0.025 p-distance universal threshold. [15,16,18,19]. Besides, the priming sites of these COI primers are prone to co-amplify other associated biota (symbionts), often yielding mixed PCR products. To separate closely related sponge taxa and avoid undesired co-amplification, molecular taxonomists supplemented the standard Folmer COI sequence of ~640 bp with a more variable downstream fragment of ~560 bp that affords expanded resolution for certain Demospongiae (the Erpenbeck’s ‘I3-M11’ extension) [18,20]. Additionally, the provision of a nuclear marker such as rDNA ITS, 18S or 28S markers [21], can significantly improve species designation [22,23,24]. By providing synchronous morphological and molecular barcoding data specimen records get catalogued and identification mistakes become less plausible. This contributes to build a robust global Porifera database. We elaborated this work under the premises of the ‘Sponge Barcoding Database’ (SBD) (http://www.spongebarcoding.org) by submitting integrated data of the sequences obtained along with morphological descriptions and pictures from the specimens (voucher and taxonomic information). SBD further interconnects GenBank (http://www.ncbi.nlm.nih.gov) for sequence information, and ‘World Porifera Database’ (http://www.marinespecies.org/porifera) where voucher information is detailed [25,26,27]. The goal of this study was to provide the first catalogue of the shallow Hawai’ian sponge fauna combining DNA barcoding and morphological descriptions, this way encouraging future studies on this biologically relevant taxon.

## Material and methods

A description of the most common shallow (0 to 15 m) sponge fauna from Kane’ohe Bay (O’ahu, Hawai’i) was carried out in a dual work. In the current study, we present the taxonomic examinations of *in situ* collections using morphological traits, microscopy and DNA barcoding. A second part of this project contrasts the relative abundance, and distribution of sponge species implementing diversity and phylogeny in different areas along a gradient of anthropogenic impact throughout the bay (unpublished data from the authors).

### Ethics statement

Experimental procedures were performed according to Hawai’i State (USA) ethics. All sponge specimens were collected under the Special Activity Permits (SAP): SAP 2014–47, SAP 2015–17, SAP 2016–55, issued by the Board of Land and Natural Resources Department, Division of Aquartic Resources, State of Hawai’i. We confirm that no further specific permissions were required, as the biological material did not involve endangered or protected species.

### Sponge collection

Sponges belonging to the most common species were collected by L. Núñez Pons in August 2014, December 2014 and February 2015 by free- and scuba-diving. Sites surveyed included nine reefs along Kane’ohe Bay (O’ahu, Hawai’i): Reefs 1 (or HIMB), 3, 5, 20, 22, 25, 42, 43 and 44 (see Fig 1). Different morphotypes of the same species were sampled in order to confirm their taxonomy. Pictures of the species were taken *in situ* and/or upon return to the Hawai’i Institute of marine Biology (HIMB) prior to processing. All samples were divided right upon collection into: ~2.5 cm^3^ pieces fixed in ethanol 80% for morphological and spicule observations, and ~0.5 cm^3^ portions preserved in ethanol absolute for down stream DNA extraction, amplification and sequencing, all stored at 4°C. Voucher specimens (~2.5 cm^3^ in 80% and ~1.5 cm^3^ in 100% ethanol) are deposited in the Gates Lab HIMB (USA) and Università Politecnica delle Marche (Italy).

### Morphological taxonomy

The spicule complement was studied according Rützler [28]. Dissociated spicules were transferred onto stubs and sputter coated with gold for SEM analyses. The skeletal architecture was examined, by optical and electronic microscopy, preparing hand-cut sections of sponge portions. SEM preparations were observed under a Philips XL 20 SEM. Measurements were obtained from 30 spicules for each spicule type and reported as smallest length—(mean ± standard deviation)—largest length x smallest width—(mean ± standard deviation)—largest width. Comparative type material was provided by several institutions and the abbreviations used in the text are as follows: Natural Museum Senckenberg, Frankfurt (SMF), National Museum of Natural History, Leiden (RMNH), Natural History Museum, London (NHM) and Bernice Pauahi Bishop Museum of Hawai’i (BPBM).

### DNA extraction, PCR and sequencing

The classical 5′- ‘Folmer’ partition of the mitochondrial metazoan marker (cytochrome c oxidase subunit I, COI) of the Barcoding of Life initiative [16], was used in combination with an additional downstream fragment, Erpenbeck’s ‘I3-M11’. This extension overlaps approximately 60bp with Folmer’s 3' COI partition and is recommended for Porifera and other diploblasts for exhibiting higher substitution rates allowing distinction down to genus level [18,20]. By combining both partitions of the COI we can retrieve a fragment of ~1,100 bp with a higher number of variable sites. The other marker sequenced was a nuclear ribosomal fragment covering the partial end of 18S rDNA, full-length of ITS1, 5.8S and ITS2, and the beginning of 28S rDNA [21].

Sponge material was extracted and total DNA was obtained from 59 specimens of the 30 represented species using two protocols: a classical C-TAP based extraction protocol on DNA-Buffer: choloform partition (adapted from lab of Dr. Andrew Baker, University of Miami), and a guanidinium based extraction established at the Gates Lab (HIMB, University of Hawai’i). For the guanidinium protocol ~0.25 cm^3^ piece of each sponge was put overnight at 4°C in 400 μl of DNA extraction buffer (50% w/v guanidinium isothiocyanate; 50 mM Tris pH 7.6; 10 μM EDTA; 4.2% w/v sarkosyl; 2.1% v/v β mercaptoethanol). Batches of 300 μL buffer-sample were incubated at 72°C for 10 min (keeping the remaining volume as backup). The resulting supernatant after centrifugation at 13,000 × rpm for 5 min was mixed with an equal volume of isopropanol and incubated at –20°C overnight. Tubes were again centrifuged at 13,000 × rpm for 15 min and the DNA pellet was washed in 70% ethanol three times, dried and resuspended (placed on ice for 1 h, and vortexing every 15 min) in Tris buffer 0.1 M pH 9. Extracted DNA was stored at -20°C until further processed. Quality and concentration of DNA were estimated on NanoDrop® ND-1000 spectrophotometer (Thermo Fisher Scientific Inc.), searching 15–150 ng/μl DNA and 260/280 and 260/230 ratios of ~1.8.

We used the primer sets, protocols and sequence target regions employed by the Sponge Barcoding Project (http://www.spongebarcoding.org/). In this manner we will contribute to the archives of taxonomic information for sponge science community. PCR amplifications were performed in 25 μl volume reactions containing 2.5 μl of 10x NH_4_ Buffer (Bioline), 0.4 μl dNTPs (10 mM), 0.8 μl BSA (20 mg/ml), 1 μl MgCl_2_ (25 mM), 0.3 μl (5U/μl) of BIOLASE™ DNA Polymerase (Bioline), 0.8 μl of each primer (10 μM), and 1.5 μl of template DNA. The standard COI partition (~640 bp) was amplified using Folmer [16] LCO1490 and HCO2198 primers. Erpenbeck’s ‘I3-M11’ extension was acquired with the primers PorCOI2fwd and PorCOI2rev, developed by Xavier et al. [20] to amplify a ~560 bp region. The fragment 18S–ITS1–5.8S–ITS2–28S (~850 bp) was amplified with the primers RA2 priming on the 3' terminus of the ribosomal small subunit, and the ITS2.2 primer targeting the 5' terminus of the large subunit, as suggested by Wörheide [19]. Thermocyling profiles of the three target markers and primer sets are presented in Table 1.

**Table 1:** Thermocycling profiles for the three target genes and primer sets of the study.

Marker	Primer set	Thermocycling conditions
COI	**LCO1490—HCO2198**	Initial denaturation: 95°C 5 min
	5'-GGTCAACAAATCATAAAGAYATYGG-3'	35 cycles (94°C 30 s, 50ºC–54°C 45 s, 72°C 90 s)
	5'-TAAACTTCAGGGTGACCAAARAAYCA-3'	Final extension: 72°C 10 min
PorCOI	**PorCOI2fwd—PorCOI2rev**	Initial denaturation: 95°C 5 min
	5'-AATATGNGGGCNCCNGGNATNAC-3'	36 cycles (94°C 30 s, 53–57°C 45 s, 70°C for 90 s)
	5'-ACTGCCCCCATNGATAAAACAT-3'	Final extension: 72°C 10 min
18S––28S	**RA2—ITS2.2**	Initial denaturation: 95°C 5 min
	5'- ACTGCCCC CATNGATAAAACAT-3'	38 cycles (94°C 30 s, 52.8°C 45 s, 72°C 60 s)
	5'-CCTGGTTAGTTTCTTTTCCTCCGC-3'	Final extension: 72ºC 10 min

DNA samples with difficult amplification were subjected to PCR optimization by regulating the concentration of additives (BSA, DMSO) and/or the thermocycling conditions. Cloning was used to separate sponge amplicons for the few PCR reactions that repeatedly yielded mixtures of different indiscernible products. The kit pGEM®-T Easy Vector System II from Promega blue-white screening was used according to manufacturer’s recommendations. Briefly, each PCR products were set up for ligation reactions with pGEM®-T Easy Vector solution and 2X Rapid Ligation Buffer T4 DNA Ligase, incubated for ~1 hr at room temperature, and then the ligation reaction was added to competent cells (JM109 Competent Cells) for transformation. Competent cells were then plated on LB/ampicillin/IPTG/X-Gal plates overnight. Eight to ten positive (white) colonies containing recombinant plasmids were picked from each plate for DNA extraction in water and PCR amplification of the inserted vector using the specific M13 forward (5´-GTAAAACGACGGCCAG-3´) and M13 reverse (5´-CAGGAAACAGCTATGAC-3´) primers.

All the molecular work was performed at the Gates Lab (HIMB), and successful amplicons of each marker region were subsequently submitted to double strand sense Sanger Sequencing on an ABI 3730xl DNA analyzer (Applied Biosystems) at the Biotech Core facilities (University of Hawai’i, Manöa Campus). Chromatograms were quality control checked, aligned and assembled on Geneious V.9.1.2 [29]. We downloaded the closest BLAST match from NCBI nucleotide database ([30]: http://www.ncbi.nlm.nih.gov/BLAST/) to confirm poriferan origin of amplified sequences. All the sequences in this study, along with the specimen descriptions, have been deposited in GenBank and Sponge Barcoding Project databases for public access. Voucher identification codes and sequence accession numbers are given in Table 1.

### Species delimitation analysis

At least three conspecific and three congeneric reference sequences were downloaded from GenBank, when available, and for species with no such reference data, we downloaded the most matching records according to BLAST searches. Reference sequences were added to our sponge alignments, and the resulting alignments were trimmed to a position at which more than 50% of the sequences had nucleotides, and missing positions at the ends were coded as missing data (Ns). To determine candidate species by molecular distinctiveness in our dataset based on the COI barcoding marker we used Automatic Barcoding Gap Discovery (ABGD) species delimitation approach [31] on the basis of Kimura 2-parameter pairwise genetic distances for identification and assignment of sequence clusters into hypothetical groups (species). All the COI alignments in fasta format were used as input files on the ABGD webpage (http://wwwabi.snv.jussieu.fr/public/abgd/abgdweb.html) and the tests showed concordant species delimitations results, with the parameters set as follows: Pmin = 0.001, Pmax = 0.1, Steps = 10, X = 1, and Nb bins = 20 (28 August 2017 version). Due to the enourmous gap positions variability in the ribosomal 18S–ITS1–5.8S–ITS2–28S fragments, we divided the dataset by genera or similarity clusters, and then ran GBlocks 0.91b independently via web interface (http://phylogeny.lirmm.fr/phylo_cgi/one_task.cgi?task_type=gblocks) for identifying and excluding blocks of ambiguous single, non-codifying gene alignments applying relaxed settings [32]. The resulting curated alignments from GBlocks could be then submitted to MEGA 6.06 [33] to compute matrices of genetic distances applying the Kimura 2-parameter model [34]. These distances were used as an additional estimate for species delimitation.

## Results

We have completed an inventory of the most common shallow Porifera at Kane’ohe Bay with a total of 65 sponge sample types collected from the surface to 15 m of depth. Most of these samples belong to different species, with a few consisting of distinct morphotypes of the same species. Traditional taxonomy and genetic data confirmed the final identification of all these specimens falling into 30 different species, 28 demosponges and 2 calcarea (see Table 2). Both, the guanadinum and the C-TAB extraction protocols provided optimal DNA concentrations (> 50 ng μL^-1^), with NanoDrop® ND-1000™ spectrophotometer measurements for A260/280 and A260/230 ranging 1.7–2.0. Nonetheless, C-TAB often afforded better results than guanidinum, producing higher yields and more consistent amplification. Overall, we retrieved good quality sequencing data in terms of sequence length (over 500bp per read), and with chromatogram traces displaying low base calling ambiguities (i.e. 11 missing bases/Ns in a 1100bp sequence = 1% ambiguity) for most 18S-ITS1-5.8S-ITS2-28S fragments (37 sequences), the 5′- ‘Folmer’ partitions (33 sequences), and the Erpenbeck’s ‘I3-M11’ extensions (26 sequences). These two last partitions of the COI marker were successfully assembled in 23 of the samples to obtain a ~1,100 bp fragment. At least one marker region was obtained from all the sponge species of our collection and all nucleotide sequences were submitted to GenBank database (see Table 2 for details and accession numbers).

**Table 2:** Sponge species collected in Kane’ohe Bay (O’ahu Island, Hawai’i).

Taxon	Collection Reef	Coordinates	Specimen voucher no.	CO-I	18S-ITS1-5.8S-ITS2-28S	SBP#
*Batzella aurantiaca*	Reef 25	21.46 N 157.82 E	HIMB_UPDM-SPO37	KY565335	KY565300	1665
*Biemna fistulosa*	Reef HIMB	21.43 N 157.79 E	HIMB_UPDM-SPO1_3	KY565306	KY565269	1666
*Callyspongia (Cladochalina)* cf. *diffusa* (purple morph)	Reef HIMB	21.43 N 157.79 E	HIMB_UPDM-SPO8	KY565312	KY565276	1667
*Callyspongia (Cladochalina) diffusa*	Reef HIMB	21.43 N 157.79 E	HIMB_UPDM-SPO16	KY565318	KY565284	1668
*Callyspongia (Cladochalina) diffusa*	Reef HIMB	21.43 N 157.79 E	HIMB_UPDM-SPO20	KY565321	KY565287	1669
*Callyspongia (Cladochalina)* sp.	Reef 22	21.46 N 157.81 E	HIMB_UPDM-SPO30	KY565329	KY565294	1670
*Callyspongia (Toxochalina)* cf. *pseudotoxa*	Reef 25	21.46 N 157.82 E	HIMB_UPDM-SPO34	KY565333	KY565297	1671
*Chondrilla mixta*	Reef HIMB	21.43 N 157.79 E	HIMB_UPDM-SPO11	KY565315	KY565280	1672
*Cladocroce burapha*	Reef 20	21.46 N 157.81 E	HIMB_UPDM-SPO29	KY565328	KY565293	1673
*Cladocroce burapha*	Reef 22	21.46 N 157.81 E	HIMB_UPDM-SPO32	KY565331	KY565295	1674
*Cliona dissimilis*	Reef HIMB	21.43 N 157.79 E	HIMB_UPDM-SPO42	KY565338	KY565301	1675
*Dysidea* cf. *arenaria*	Reef 25	21.46 N 157.82 E	HIMB_UPDM-SPO39	–	KY565304	1676
*Dysidea* sp. 1	Reef HIMB	21.43 N 157.79 E	HIMB_UPDM-SPO12	KY565320	KY565281	1677
*Dysidea* sp. 1	Reef HIMB	21.43 N 157.79 E	HIMB_UPDM-SPO18	KY565319	KY565286	1678
*Dysidea* sp. 2	Reef HIMB	21.43 N 157.79 E	HIMB_UPDM-SPO17	KY565316	KY565285	1679
*Echinodictyum asperum*	Reef HIMB	21.43 N 157.79 E	HIMB_UPDM-SPO41	KY565337	KY565303	1680
*Gelliodes* sp.	Reef HIMB	21.43 N 157.79 E	HIMB_UPDM-SPO24	KY565325	–	1681
*Gelliodes wilsoni*	Reef HIMB	21.43 N 157.79 E	HIMB_UPDM-SPO10	KY565314	KY565278 / KY565279	1682
*Gelliodes wilsoni*	Reef 20	21.46 N 157.81 E	HIMB_UPDM-SPO28	KY565327	KY565292	1683
*Haliclona (Reniera) aquaeductus sensu* de Laubenfels 1951	Reef 25	21.46 N 157.82 E	HIMB_UPDM-SPO40	KY565336	KY565302	1684
*Haliclona (Soestella) caerulea*	Reef HIMB	21.43 N 157.79 E	HIMB_UPDM-SPO21	KY565322	KY565288	1685
*Haliclona (Soestella) caerulea*	Reef HIMB	21.43 N 157.79 E	HIMB_UPDM-SPO23	KY565324	KY565290	1686
*Haliclona (Soestella) caerulea*	Reef 22	21.46 N 157.81 E	HIMB_UPDM-SPO31	KY565330	–	1687
*Hymeniacidon chloris*	Reef HIMB	21.43 N 157.79 E	HIMB_UPDM-SPO22	KY565323	KY565289	1688
*Hymeniacidon gracilis*	Reef 44	21.48 N 157.83 E	HIMB_UPDM-SPO26_27	KY565326	KY565291	1689
*Iotrochota baculifera*	Reef HIMB	21.43 N 157.79 E	HIMB_UPDM-SPO13_45	KY565317	KY565282	1690
*Leucetta* sp.	Reef 25	21.46 N 157.82 E	HIMB_UPDM-SPO35	–	KY565298	1691
Leucosolenida	Reef HIMB	21.43 N 157.79 E	HIMB_UPDM-SPO14	–	KY565283	1692
*Lissodendoryx (Waldoschmittia) hawaiiana*	Reef HIMB	21.43 N 157.79 E	HIMB_UPDM-SPO5	KY565309	KY565273	1693
*Monanchora clathrata*	Reef HIMB	21.43 N 157.79 E	HIMB_UPDM-SPO7_38	KY565311	KY565275	1694
*Mycale (M*.*) grandis*	Reef HIMB	21.43 N 157.79 E	HIMB_UPDM-SPO4_19_43_44	KY565308	KY565271 / KY565272	1695
*Mycale (Zygomycale) parishii*	Reef 25	21.46 N 157.82 E	HIMB_UPDM-SPO33	KY565332	KY565296	1696
*Pseudoceratina purpurea*	Reef HIMB	21.43 N 157.79 E	HIMB_UPDM-SPO9	KY565313	KY565277	1701
*Spheciospongia solida*	Reef HIMB	21.43 N 157.79 E	HIMB_UPDM-SPO2_25	KY565307	KY565270	1697
*Suberites diversicolor*	Reef HIMB	21.43 N 157.79 E	HIMB_UPDM-SPO46	–	KY565305	1698
*Tedania (Tedania) ignis*	Reef HIMB	21.43 N 157.79 E	HIMB_UPDM-SPO6	KY565310	KY565274	1699
*Tedania (Tedania) ignis*	Reef 25	21.46 N 157.82 E	HIMB_UPDM-SPO36	KY565334	KY565299	1700

Museum voucher identification numbers of the specimens, GenBank and Sponge Barcoding Project (SBP#) accession numbers of submitted COI and 18S–ITS1–5.8S–ITS2-28S sequences. (–) means that no sequence was successfully obtained. HIMB (Hawai’i Institute of Marine Biology); UPDM (Università Politecnica delle Marche).

The recursive ABGD analysis based on the COI marker with the Erpenbeck’s ‘I3-M11’ extension (1,100 bp) identified 81 groups–comprising our sponge dataset and reference sequences–given a series of prior values from 0.001 to 0.046, whereas the initial partition yielded 65 groups (S1 Fig). Analysis with only Folmer’s COI partitions (621 bp) produced 67 and 61 groups in the partitions. The groups from the recursive partition of the 1,001 bp COIs were the most consistent with our designated morphospecies except for some conspecifics in *Monanchora*, *Iotrochota*, *Chondrilla*, *Pseudoceratina* and *Tedania*. In the full data set, intraspecific distances ranged from 0% to 2.3% and congeneric interspecific distances from 0.5% to 15.3%. The mean K80 Kimura intraspecific distances were: 2% for *Monanchora clathrata*, 0.3% *Iotrochota baculifera* and 1.3% for *Tedania ignis*, 0.0% for *Biemna fistulosa*, 0.0% for *Cladochalina (Cladochalina) diffusa*, 0.0% for *Cladocroce burapha*, and 0.3% for *Haliclona (Soestella) caerulea*. The mean congeneric interspecific Kimura distances were: 6.4% for genus *Hymeniacidon*, 4.4% for *Spheciospongia*, 5% for *Cliona*, 5.4% for *Biemna*, 2% for *Monanchora*, 5.8% for *Lissodendoryx*, 0.7% for *Iotrochota*, 7% for *Mycale*, 1.9% for *Tedania*, 4.1% for *Bienma*, 3.8% for *Echinodictyum*, 6.8% for *Callyspongia*, 9.5% for *Haliclona*, 5.3% for *Dysidea*, 0.6% for *Chondrilla*, and 0.8% for *Pseudoceratina*. For genera *Batzella*, *Cladocroce* and *Gelloides* there were no reference congeneric nucleotide entries, so distance estimations were not feasible. The groups resulting from ABGD analyses and the MEGA p distance matrices were used in the assignment of sequences as members of hypothetical clusters, and check for species delimitation and congruence with morphological identifications for each specimen of this collection (see S1 and S2 Tables for pairwise distance matrices).

Systematic information with detailed morphological and spicule descriptions, and with DNA-barcoding remarks are provided in the next sections. The classification used followed the Systema Porifera [22] and the recent revision proposed by Morrow & Cárdenas [35]. Species with sampling localities and coordinates information is summarized in Table 2.

### Systematics

Class: Calcarea

     Subclass: Calcaronea

          Order: Leucosolenida

          Order: Clathrinida

               Family: Leucettidae

                    Genus: *Leucetta*

Class: Demospongiae

     Subclass: Heteroscleromorpha

          Order: Suberitida

               Family: Halichondriidae

                    Genus: *Hymeniacidon*

                         *Hymeniacidon chloris* de Laubenfels, 1950

                         *Hymeniacidon gracilis* (Hentschel, 1912)

          Family: Suberitidae Schmidt, 1870

               Genus: *Suberites*

                    *Suberites diversicolor* Becking & Lim, 2009

          Order: Clionaida

               Family: Clionaidae

                    Genus: *Cliona*

                         *Cliona dissimilis* Ridley & Dendy, 1886

                         *Spheciospongia solida* (Ridley & Dendy, 1886)

          Order: Poecilosclerida

               Family Chondropsidae

                    Genus: *Batzella*

                         *Batzella aurantiaca* (Lévi, 1958)

               Family Coelosphaeridae

                    Genus: *Lissodendoryx*

                         *Lissodendoryx (Waldoschmittia) hawaiiana* de Laubenfels, 1950

               Family: Crambeidae

                    Genus: *Monanchora*

                         *Monanchora clathrata* Carter, 1883

               Family: Iotrochotidae

                    Genus: *Iotrochota*

                         *Iotrochota baculifera* Ridley, 1884

               Family Mycalidae

                    Genus: *Mycale*

                          *Mycale (Mycale) grandis* Gray, 1867

                         *Mycale (Zygomycale) parishii* (Bowerbank, 1875)

               Family: Tedanidae

                    Genus: *Tedania*

                         *Tedania (Tedania) ignis* (Duchassaing & Michelotti, 1864)

          Order: Biemnida

               Family: Biemnidae

                    Genus: *Biemna*

                         *Biemna fistulosa* (Topsent, 1897)

          Order: Axinellida

               Family: Raspailiidae

                         Genus: *Echinodictyum*

                              *Echinodictyum asperum* Ridley & Dendy, 1886

          Order: Haplosclerida

               Family: Callyspongiidae

                    Genus: *Callyspongia*

                         *Callyspongia (Cladochalina) diffusa* (Ridley, 1884)

                         *Callyspongia (Cladochalina)* sp.

                         *Callyspongia (Toxochalina)* cf. *pseudotoxa* Muricy & Ribeiro, 1999

               Family: Chalinidae

                    Genus: *Cladocroce*

                         *Cladocroce burapha* Putchakarn, de Weerdt, Sonchaeng & Van Soest, 2004

                    Genus: *Haliclona*

                         *Haliclona (Soestella) caerulea* (Hechtel, 1965)

                         *Haliclona (Reniera)* cf. *aquaeductus* (Schmidt, 1862) *sensu* de Laubenfels, 1951

               Family: Niphatidae

                    Genus: *Gelliodes*

                         *Gelliodes wilsoni* Carballo, Aquilar-Camacho, Knapp & Bell, 2013

                         *Gelliodes* sp.

     Subclass: Keratosa

          Order: Dictyoceratida

               Family: Dysideidae

                    Genus: *Dysidea*

                         *Dysidea* cf. *arenaria* Bergquist, 1965

                         *Dysidea* sp. 1

                         *Dysidea* sp. 2

     Subclass: Verongimorpha

          Order: Chondrillida

               Family: Chondrillidae

                    Genus: *Chondrilla*

                         *Chondrilla mixta* Schulze, 1877

          Order: Verongiida

               Family: Pseudoceratinidae

                    Genus: *Pseudoceratina*

*Pseudoceratina purpurea* (Carter, 1880)

Class Calcarea

Subclass Calcaronea

Order Leucosolenida

Fig 2A

**Fig 2:**
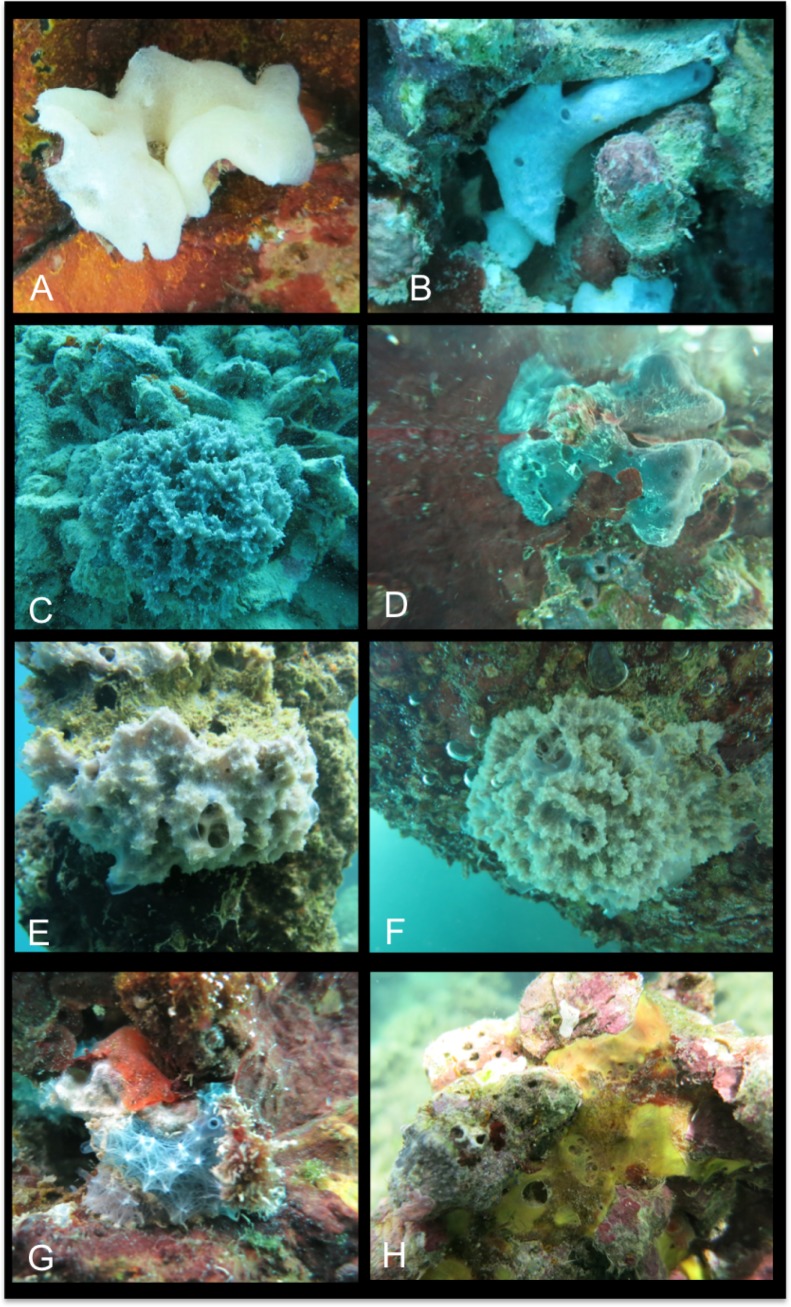
Sponge specimens not identified at the species levels. A Leucosolenida; B *Leucetta* sp.; C *Callyspongia (Cladochalina)* sp.; D *Gelliodes* sp.; E *Dysidea* sp. 1, SPO12; F *Dysidea* sp. 1, SPO18; G *Dysidea* sp. 2.

Material: SPO14, Reef HIMB (Lagoon Floating Deck), 0.2 m.

Description: White calcareous sponge of crisp texture, lobulate in shape with grooves and folds and displaying conspicuous oscules. The sponges consisted in small individuals ~3 cm^3^ encrusting a floating structure.

Spicules: Big equiangular triactines with straight actines up to 1500 μm long; small equiangular triactines with straight actines up to 180 μm long. Sagittal tetractines often with curved actines about 150–350 μm long.

Remarks: Calcareous sponges are characterized by a highly unusual mitocondrial genome formed of multiple linear chromosomes, modifications in genetic code, fragmented rRNA genes, tRNA editing, and high rate of evolution [36]. PCR reactions were for some unknown cause not successful in amplifying any partition of the COI region, and thus no reliable barcoding COI distance analysis was done for this sample. Instead, the ribosomal fragment obtained was a sequence spanning partial 18S, ITS-1 and partial 5.8S, which showed the closest similarity (89.6%) with a calcareous sponge *Sycon ancora* from the Adriatic Sea, Italy. Morphologically, *Sycon ancora* differs from our specimen for its general morphology (vase-shaped) and for its spicular features (anchor-like tetractines and diactines).

Subclass Calcinea

Order Clathrinida

Family Leucettidae de Laubenfels, 1936

*Leucetta* sp.

Fig 2B

Material: SPO35, Reef 25, 3 m.

Description: Bright light blue sponge with iridescent tones. The texture is rigid calcareous and rounded in shape. Specimens turned light beige in alcohol. Individuals were ~10 cm long and have evident oscules. They were found growing in crevices or scarves.

Spicules: Big equiangular triactines with straight actines up to 1600 μm long; medium-sized equiangular triactines with actines about 300 μm long; and small equiangular triactines with actines about 25–60 μm long.

Remarks: Sponge matching Leucettidae [37]. Sequencing data from the COI marker were not possible to retrieve. The target ribosomal fragment was fully successfully obtained (18S-ITS1-5.8S-ITS2-28S), and matched within the species *Leucetta microraphis*, revealing 99.7% similarity with a clone from Wistari Reef (Great Barrier Reef, Australia), and 98.8% with another *L*. *microraphis* from Saudi Arabia (Red Sea). Instead, p distances with other available congeneric *Leucetta* references ranged 3.8% to 13.1% divergence. From a morphological point of view the Hawai’ian specimen differs from *L*. *microraphis* in the general morphology and color; *L*. *microraphis* is dark pink, brownish pink or pinkish-white and variable in shape (lobate, flabellate or massive), with oscules and atrial lumen up to 2 cm in diameter [38]. Moreover the big and small triactines are longer (up to 1980 μm and 150 μm, respectively) in *L*. *microrhapis*. The tripods present in Henckel’s species, even if rare [38] were not found in our sample.

Class Demospongiae

Subclass Heteroscleromorpha

Order Suberitida

Family Halichondriidae Gray, 1867

*Hymeniacidon chloris* de Laubenfels, 1950

*Hymeniacidon chloris* de Laubenfels, 1950: 27 [4].

Material: SPO22, Coconut Is. Lagoon Floating Deck, 0.2 m.

**Fig 3:**
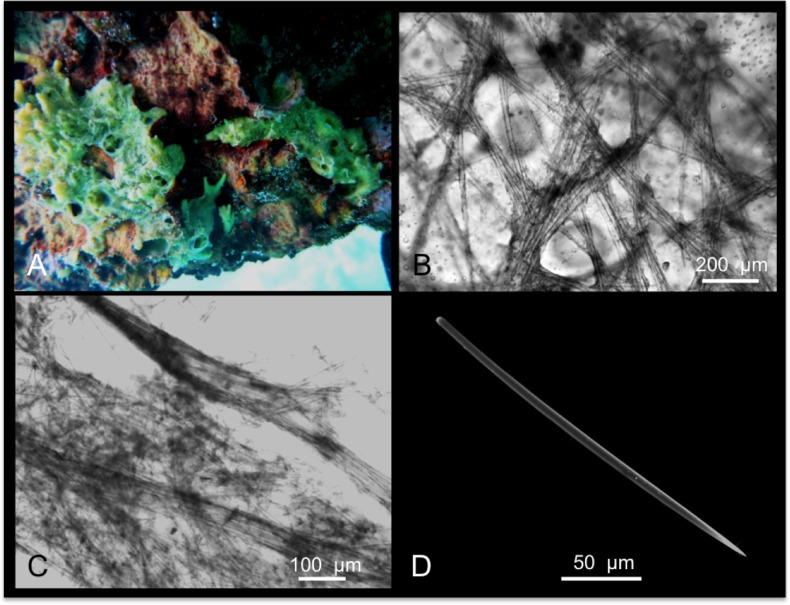
Hymeniacidon chloris. A alive specimen; B intercrossing bundles of the ectosomal skeleton; C choanosomal skeleton; D Style.

Description: Massive encrusting sponge with irregular surface and numerous thin tapering projections (Fig 3A). The colour of the living specimen is yellowish, greenish; the preserved specimen becomes light brown to light buff. The material is soft and spongy.

Skeleton: The ectosomal skeleton is fleshy membranous with spicules tangentially arranged and organized in thin intercrossing bundles (Fig 3B). In the choanosome, styles are irregularly arranged, but it is possible to recognize tracts of spicules running towards the surface (Fig 3C).

Spicules: Styles (Fig 3D) are slightly curved, and have a wide size range of 220-(365.3±112.3)-515 x 3.6-(8.1±3.7)-14.4 μm.

Distribution: Hawai’i.

Remarks and discussion: This species was exclusively known from its type locality in Kane’ohe Bay, Moku O Loe [4]. Our sequences are the first barcoding data of COI and ribosomal genes available for *H*. *chloris*. This specimen seems well delimited as species according to COI genetic p distances, revealing 5.6–9.9% divergence with five available congeneric reference sequences (*H*. *flavia*, *H*. *perlevis*, two *H*. *sinapium*, *H*. *heliophila*), and 15.3% against *H*. *gracilis* (SPO26) from this Hawai’ian dataset. The ribosomal region also showed significant divergence with available congeneric reference sequences, p distances ranging from 19.7% against a *Hymeniacidon* sp. and up to 45.4% with *H*. *gracilis* (SPO26).

*Hymeniacidon gracilis* (Hentschel, 1912)

Fig 4

**Fig 4:**
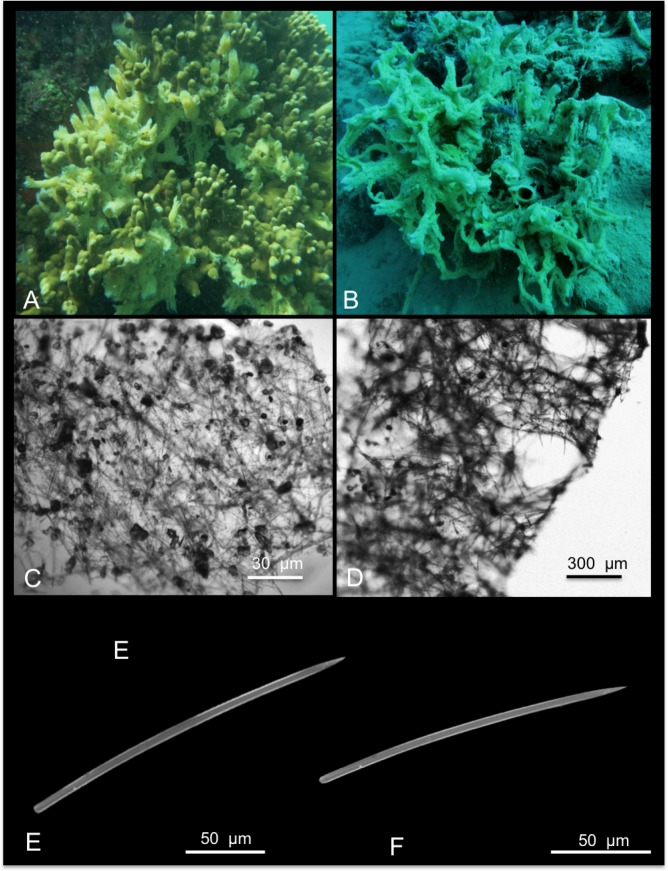
Hymeniacidon gracilis. A, B alive specimens; C tangential, intercrossing spicules of the ectosomal skeleton; D choanosomal skeleton; E, F styles.

*Stylotella digitata* var. *gracilis* Hentschel, 1912: 356 [39]; Hooper et al., 1997: 55 [40].

Material: SPO26, Reef 44, 5 m; SPO27, Reef 20, 5 m.

Other material: SMF 970 Sintype.

Description: Massively encrusting sponge growing on sand and on coral rocks and rubble. The surface is irregular with digitate fistules opening in oscules cavities (Fig 4A and 4B). In the specimen SPO 27 the surface was also characterized by numerous branching long projections (Fig 4B). The colour of the living specimens is light yellow-cream (Fig 4A and 4B), and turns cream in alcohol. The texture is soft and fragile.

Skeleton: The ectosomal skeleton is made by tangential intercrossing spicules. Numerous foreign spicules and sand grains are present (Fig 4C). In the choanosomal skeleton, spicules are randomly arranged, but in the superficial parts they tend to form ascending tracts of about 3/4 spicules connected by transversal spicules, this way creating a pseudo-organized reticulate skeleton (Fig 4D).

Spicules: Styles are of a single category, in general straight (Fig 4E and 4F); their width is constant along the spicule axis, and the tips are sharply pointed.

Distribution: This species is known from Indonesia (Aru island) [39] and from Australia (Darwin Harbour) [40]. It is a new record for the Hawai’ian archipelago.

Remarks and discussion: The Hawai’ian specimen fits with the species described by Hentshel [39] and later by Hooper et al. [40] in what regards the general external morphology, shape and size of styles (respectively 224–256 x 4–5 μm and 218–285 x 2–8 μm; see also Table 3). Re-examination of the holotype confirmed the specific determination. In this study we provide the first barcoding data of this species for COI and ribosomal genes. This sponge clearly diverged from the five mentioned congeneric *Hymeniacidon* references (see previous species description above), with distance percentages ranging 9.7% to 13.5% for the COI marker, and 45.4% to 52.9% for the ribosomal 18S-ITS1-5.8S-ITS2-28S fragment.

**Table 3:** Spicule measurements of *Hymeniacidon gracilis*.

	Styles (μm)
SPO 26	195-(215±9)-230 x 3.7-(5.6±1.3)-7.5
SPO 27	175-(206.9±12.9)-230 x 2.5-(4.4±0.8)-5
Sintype (SMF 970)	200-(233.3±18.3)-260 x 3.7-(6.3±1.7)-8.7

Family Suberitidae Schmidt, 1870

*Suberites diversicolor* Becking & Lim, 2009

Fig 5

**Fig 5:**
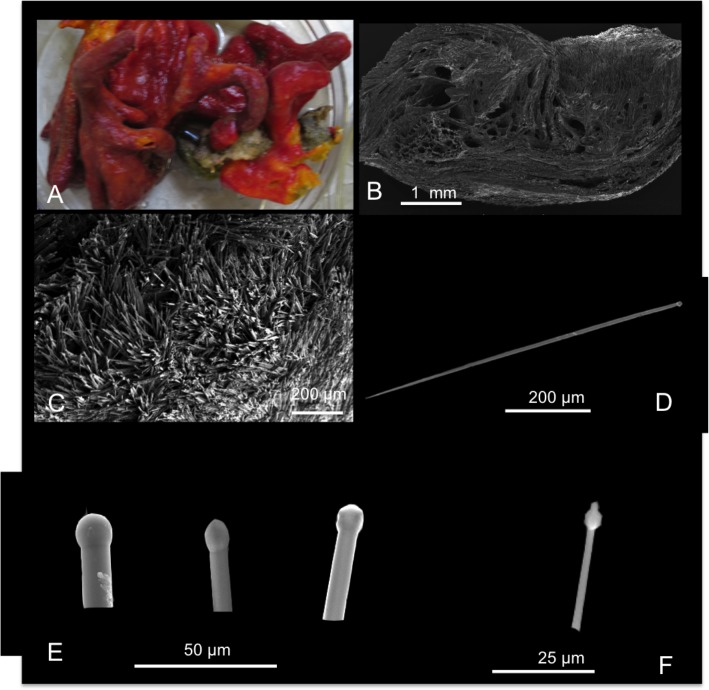
Suberites diversicolor. A fresh collected specimen; B SEM photograph of a digitate projection showing the cavernous structure; C microhispid surface; D tylostyle; E, F magnification of the tylostyle heads.

*Suberites diversicolor* Becking & Lim, 2009: 855 [41].

Material: SPO 46, Coconut Is., Floating Deck, 0.5 m.

Other material: RMNH Por. 4672 Holotype.

Description: The sponge grows massively on the base attached to the substrate, from which numerous digitate and ramose projections develop (Fig 5A). The projections may be long up to 10 cm, approximately, and with rounded terminal ends. The studied specimens were red externally and yellow inside (Fig 5A), and there were other collected specimens with outer dark green coloration that were similarly yellow internally. The surface is microhispid and papillate. The sponge is cavernous and its consistency is spongy and elastic (Fig 5B).

Skeleton: In the periphery, smaller tylostyles are concentrated to form brushes; in the internal part, spicules form vague and dense tracts that are directed outwards creating a microhispid surface (Fig 5C).

Spicules: Tylostyles (Fig 5D), straight with sharp tips, in a single, wide size range 160-(426.4±220.1)-750 x 2.5-(5.6±2.8)-11.2 μm; their head is rounded (Fig 5E), often trilobated (Fig 5F).

Distribution: Indonesia, India, Singapore, Vietnam, Northern Australia [41] and Hawai’i.

Remarks and discussion: The sample from Hawai’i fits with the species *S*. *diversicolor* according to the body shape, color, skeletal arrangement, and spicule shape and size. This species shows a very high phenotypical plasticity (in shape, colour, surface and spicule size), often linked to the type of habitat [41]. The sample in Becking & Lim ([41]: 856, Fig 2D) is more similar to the Hawai’ian specimens, which have generally smooth surface and few small papillae. The skeleton in the analysed specimen is denser than in *S*. *diversicolor*, but the general organization (typical of the genus) does not differ.

De Laubenfels ([4]: 28) reports the presence of *Terpios zeteki* = *Suberites zeteki* in Kane’ohe Bay (Hawai’i). This species is now considered a junior synonymous of *S*. *aurantiacus* [42, 43,44]. The same author also claimed that this species was very common throughout Hawai’i [6].

*S*. *aurantiacus* has a native range covering the Caribbean and west coast of Panama, and it is reported by de Laubenfels [37] in the Pacific end of the Panama Canal (Table 4). On the base of these data this species has been identified as *S*. *zeteki* and was introduced in Hawai’i. It is now common in harbours, and in Kane’ohe Bay it is found on floating docks, and also on hulls of ships [3].

**Table 4:** Comparison of the tylostyles and of other morphological characteristics among *S*. *diversicolor*, *S*. *aurantiacus* and its synonimies.

	Colour	Shape	Ectosomal skeleton	Choanosomal skeleton	Tylostyle length (μm)	Tylostyle width (μm)	Reference	Distribution
*Terpios fugax*	internal yellow; external blue or yellow	rounded, lobated, encrusting	radiate cluster or bouquets of tytlostyles		160–400	2–3	De Laubenfels [45]: 103	Bermuda
*Suberites zeteki*	ochre-yellow; at the surface greenish or reddish	massive, with digitate projections	tylostyles densely packed in the ectosome		up to 700 x	3–20	De Laubenfels,[37]: 450	Panama canal, Pacific coast
*Suberites aurantiacus*			brusches of a small class of tylostyles		700 and 150	20 and 7.5	Rützler & Smith [44]: 390	Pacific coast of Panama (holotype revision), Virgin Islands, Puerto Rico, Bermuda, Belize, Brazil, Gulf of Mexico
*Terpios zeteki*	internal dull or orange yellow internally, superficial color variable: bluish green, orange-yellow, carmine to orange				130–750	2.5–19	Pulitzer-Finali [46]: 88	Jamaica, Puerto Rico
*Suberites diversicolor*	purple, olive green, blue, orange	massive with globular branches, encrusting	smaller tylostyles directed outwards in palisade	densely packed tylostyles in vague tracts and/or in confusion	165-(499)-810 (Holotype)	2.5-(8.9)-17.5	Becking & Lim [41]: 855	Indonesia Borneo, Australia
*Suberites diversicolor*	red, orange outside and yellow inside; green outside and yellow inside	brunched, digitated internally cavernous	smaller tylostyles form brushes	tylostyles in vague and dense tracts	105–760	2–10	This paper	Hawai’i

According to morphological data (shape and size of spicules, skeletal arrangement, sponge shape and external features) *S*. *aurantiacus*, *S*. *diversicolor* and specimens from Hawai’i are not clearly separable (Table 4). DeFelice et al. [3] in fact, examined the holotype and considered the Hawai’ian specimens to be conspecific with the Caribbean species. Previous comparisons of standard 534 bp partitions of COI sequences further showed a great genetic similarity indicating that *S*. *diversicolor* differed only by 1% to *Suberites aurantiacus* [41]. We were not successful obtaining suitable sequencing data for the COI region. Various ribosomal marker sequences are also available in NCBI database, including several 18S-ITS1-5.8S-ITS.2-28S and 18S nucleotide entries from *S*. *diversicolor*, as well as some *S*. *aurantiacus* 18S and 28S (no ITS). We retrieved a ribosomal fragments spaning ITS1-5.8S-ITS.2-28S (partial), which revealed exact match (100% similarity) after trimming and gap curation with reference *S*. *diversicolor* from Indonesia. Identical match was also found against the last 75bp segment of the 18S from a Panamenian *S*. *aurantiacus*. Nonetheless, the alignment with this fragment–not covering the ITSs–was too short (~15%) to consider such comparison as valid. Striking large p distance values (66.3%) clearly separated our specimens from other congeneric reference sequences, e.g., *S*. *domuncula* and *S*. *ficus*, These results agree with the morphological findings previously discussed, and keep suggesting potential synonymy between *S*. *aurantiacus* and *S*. *diversicolor*.

Order Clionaida

Family Clionaidae d'Orbigny, 1851

*Cliona dissimilis* Ridley & Dendy, 1886

Fig 6

**Fig 6:**
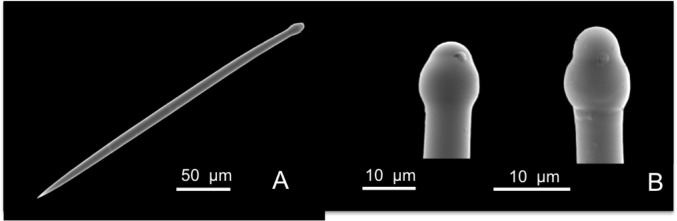
Cliona dissimilis. A tylostyle; B magnification of the tylostyle head.

*Cliona dissimilis* Ridley & Dendy, 1886: 490 [47]; Fromont et al., 2005: 154 [48].

Material: SPO 42, Reef 1 HIMB, 1.5 m.

Description: Boring sponges in *beta* stage found on dead shells and coral substrates of the genus *Porites* and *Montipora* and calcareous rubble. The sponge was covered with a thin layer of tissue of ~2 mm, and is red-orange alive to dark orange, turning brownish ochraceous in alcohol. In the preserved sample, inhalant and exhalant papillae up to 2 mm high and about 2–4 mm in diameter are visible. The sponge excavates the substrate without making clear erosion chambers, and creates irregular erosion channels up to 3 mm large.

Skeleton: In the external layer covering the coral, tylostyles are perpendicularly arranged to the surface with the heads in contact with the substrate; in the papillae the tylostyles are closely packed. Inside the substrate tylostyles are irregularly arranged.

Spicules: Tylostyles in general straight or slightly curved (Fig 6A), with the vesicle in the tyle and the axial filament visible; they measure 250-(291.3±19.6)-320 x 7.5-(8.7±1.4)-12.5 μm, and have oval heads that are trilobated (Fig 6B). Tips are hastate.

Distribution: It is the first record for Hawai’i; the sponge is known for New Guinea, Indonesia and Australia [48].

Remarks and discussion: The sample from Hawai’i fits with the original description of the species (see [47,49]) for the shape and size of the chambers and for the general organization. Spicules are also comparable to this holotype for their shape and size. Ridley & Dendy [49] described *C*. *dissimilis* as *beta* stage sponges boring and encrusting corals, with oscules and ostia confined to different layers of the laminar corals. The skeleton is organized similar as in other *Cliona* species. Tylostyles are rather slender, with very well marked heads (320 x 6.5 μm). In 2005, Formont et al. recorded the species for the first time in Australia. They described it as orange (alive) excavating relatively large cavities (1–2 mm), and with tylostyles displaying an oval tyle with the axial vesicle always present. The sequences submitted in this study (COI and ribosomal) are the first barcoding data available for *C*. *dissimilis*. According to genetic distance analyses our material was clearly distinct from COI reference sequences representing eight congeneric sponges available in GenBank (*Cliona* sp., *C*. *celata*, *C*. *chilensis*, *C*. *delitrix*, *C*. *jullieni*, *C*. *orientalis*, *C*. *vermifera* and *C*. *viridis*) with p distances ranging 4.5% to 12.8%. The ribosomal fragment also showed compelling species delimitation (5.6% - 35.6% dissimilarity) against 11 available specific references (*Cliona aprica*, *C*. *caribbaea*, *C*. *celata*, *C*. *chilensis*, *C*. *tenuis*, *C*. *varians*, *C*. *californiana*, *C*. *laticavicola*, *C*. *orientalis*, *C*. *vermifera* and *C*. *viridis*).

*Spheciospongia solida* (Ridley & Dendy, 1886)

Fig 7

**Fig 7:**
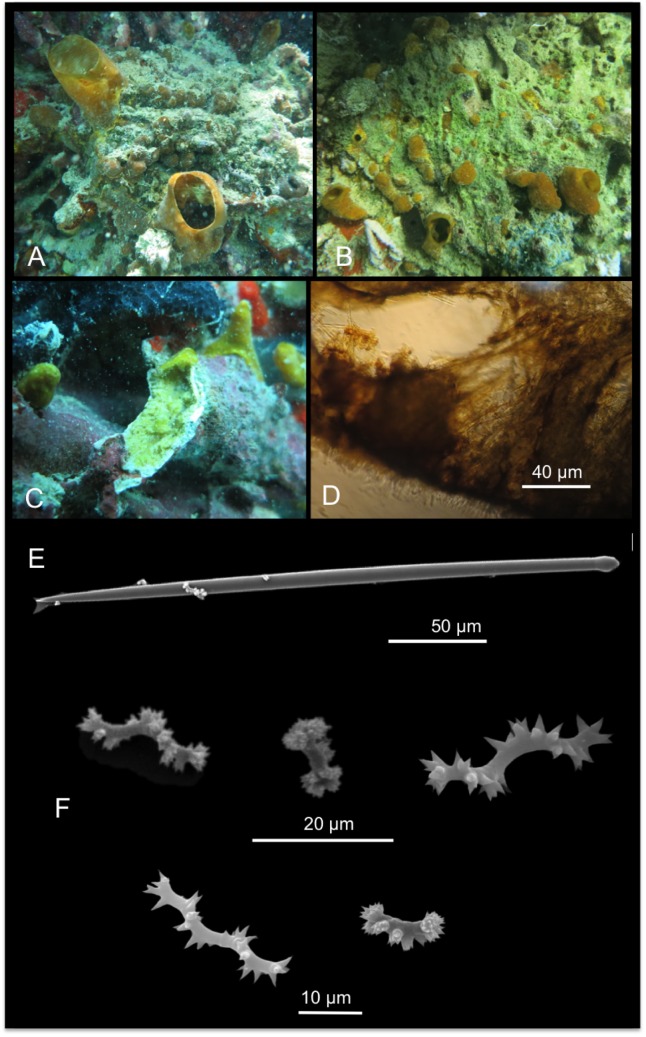
Spheciospongia solida. A-C alive specimens; D transversal section of the sponge skeleton, shoving tylostyle hispidation; E tylostyle; F different kind of spirasters.

*Spirastrella solida* Ridley & Dendy, 1886: 491 [47]; Ridley & Dendy, 1887: 231[49].

Material: SPO2, Coconut Is. Point Lab, 5 m; SPO25, reef 44, 5 m.

Description: Boring sponges excavating on dead coral and rubble, and on basal parts of living colonies (genera *Porites* and *Montipora*). The specimens exhibit inhalant, closed, often gathered fistules, and large exhalant fistules (Fig 7A–7C). The coloration is brown, yellowish (Fig 7A–7C) in part given by a rich *Symbiodinium* (Zooxanthellae) population living symbiotically within the sponge pinacoderm and choanosome, observed as brownish rounded 6–13 μm cells. The examined portions are small parts of fistules up to 3 x 1.5 cm long. Surface is microhispid under microscope observation. The basal parts of the sponge, in contact with the substrate, are full of bored material engulfed in the tissue. The sponge is firm and hard.

Skeleton: The choanosomal skeleton is compact and confused in the internal part. In the peripherical parts, tracts of tylostyles running toward the surface are detectable. Tips of the spicules hispid the surface (Fig 7D). Microscleres are concentrated in the external parts creating a crust of spirasters.

Spicules: Tylostyles in general straight, slightly curved (Fig 7E); heads from rounded to ovoid. Spirasters are variable in shape (Fig 7F): there are spirasters with one bend and spines in bouquets at the extremities and along the convex parts, and also spirasters with three or four bends often with simple conical spines (measurements are shown in Table 5).

**Table 5:** Spicule measurements of *Spheciospongia solida*.

	Tylostyles (μm)	Spirasters (μm)
SPO 2	230-(461.7±130.8)-770 x 5-(10.9±3.5)-16.2	10-(15±7.2)-37.5 x 2.5
SPO 25	340-(521.5±91.5)-650 x 10-(13±4.7)-20	10-(15.6±8.2)-42.5 x 2.5

Distribution: Described in the Philippines [47], also recorded in Indonesia (Ambon [50,51,52]), in Vietnam [53] and Thailand [54]. It has been recently recorded in Northwestern Hawai’ian Islands [55].

Remarks and discussion: Longer spirasters illustrated by Ridley and Dendy ([49], PL XLV Fig 13E) are rare, but Hawai’ian specimens fit with the species *S*. *solida*. The sponge was described as “lobate or digitate; consisting of a broad base, containing a large amount of embedded foreign matter, from which arise broad, fleshy looking lobes, the larger of which have each one osculum” [49]. The studied specimens from Hawai’i are excavating into corals producing large cavities (Fig 6C); they probably correspond to sponges in the early growth stage as pointed by Rützler [56]. Here we provide the first barcoding records for COI and ribosomal genes for this sponge species. The COI sequence revealed that the genetic distinctiveness from a clone of *S*. *vesparium* from Vietnam was 1.1%, which is lower than the accepted threshold for species delimitation (2.5%), whereas the distance against a reference *S*. *vagabunda* from the Red Sea was 6.1%. This could be another case group in which the COI is not resolutive enough. However, we suspect that the reference from the Vietmanese *S*. *vesparium* might actually correspond to a misclassified *S*. *solida*, as *S*. *vesparium* is normally distributed along the Caribbean and North Brasil. The existence of further mistakes in the databases is not discarded, especially considering the recent reorganization of the previously assigned genus *Spirastrella*. There were no available *Spheciospongia* ribosomal ITS sequences to compare with our dataset, the closest *Spirastrella* references though showed 79% similarity.

Order Poecilosclerida

Family Chondropsidae

*Batzella aurantiaca* (Lévi, 1958)

Fig 8

**Fig 8:**
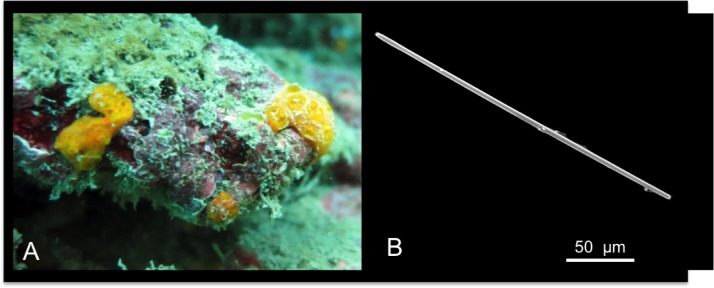
Batzella aurantiaca. A alive specimen. B strongyle.

*Prianos aurantiaca* Lévi, 1958: 33 [57].

Material: SPO 37, Reef 44, 4.5 m.

Description: Small yellow-orange sponge growing in patches, and encrusting dead coral substrate, crustose algae and crevices (Fig 8A). Individuals are very thin and fragile, with a surface characterized by numerous cribrous areas.

Skeleton: Strongyles make small tracts or bundles in the choanosome.

Spicules: Only thin and straight strongyles (Fig 8B); they measure 125-(190.4±23.4)-225 x 2.5-(3.1±1.1)-5μm.

Distribution: Red Sea [57,12] and Hawai’i (O’ahu Island).

Remarks and discussion: The specimen SPO 37 fits with the descriptions by Lévi [57], and later by Calcinai et al. [12] who reported for the first time this species in Hawai’i, growing on a colony of *Carijoa riisei*. As already pointed out by the former authors, *Batzella aurantiaca* may be a new introduction to Hawai’i, or it may have been overlooked by previous studies. Population studies employing molecular approaches could clarify its cryptogenic nature. For the moment the only sequencing data available are given in the present study. We were successful in amplifying the ribosomal fragment, however for the COI marker we could only retrieve the Folmer standard partition, the Erpenbeck’s ‘I3-M11’ extension could not be obtained. No validation analysis regarding species delimitation could be included, as there are no congeneric reference sequences available in GenBank.

Family losphaeridae Dendy, 1922

*Lissodendoryx (Waldoschmittia) hawaiiana* de Laubenfels, 1950a

Fig 9

**Fig 9:**
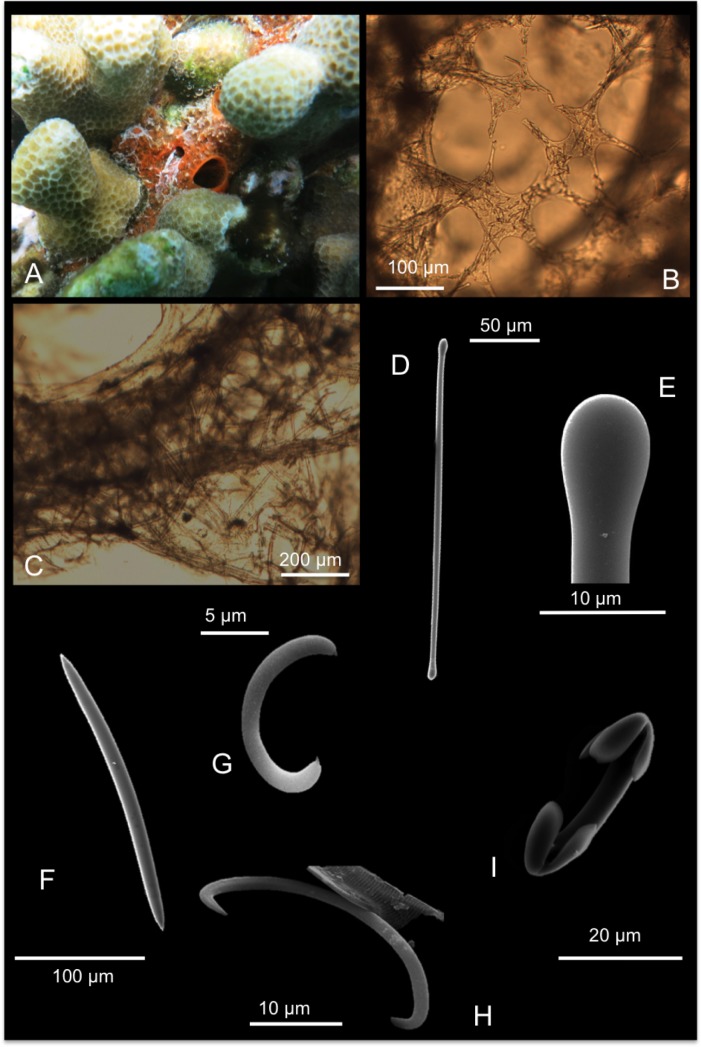
Lissodendoryx (Waldoschmittia) hawaiiana. A alive specimen; B ectosomal, dermal membrane; C choanosomal skeleton; D tylote; E tylote head magnification; F oxea; G sigma I with small denticle; H sigma II; I arcuate chela.

*Damiriana hawaiiana* de Laubenfels, 1950a: 50 [4].

Material: SPO 5, Coconut Is. Point Lab, 3 m;

Description: Massively encrusting sponge growing within crevices of *Porites* colonies. The sponge is bright orange and with the surface covered by numerous cribrous areas (Fig 9A). Light orange when preserved. Large oscules are evident. The consistence is spongy. The sponge produces copious mucous secretions when handled.

Skeleton: An ectosomal dermal membrane pierced by cribrous ostial areas supports tangential tylotes and microscleres (Fig 9B). Beneath, few scattered funs of tylotes are detectable; the choanosomal skeleton is made by oxeas making a reticulated skeleton with ascending tracts of oxeas (about 50 μm) and meshes in between (Fig 9C).

Spicules: Ectosomal tylotes, straight and smooth (Fig 9D and 9E) 215-(228±9.2)-245 x 3.7-(5.8±1.1)-7.5 μm; oxeas slightly curved with acerate tips 255-(271.3±9.2)-285 x 10-(13±2.5)-15 μm (Fig 9F); sigmas I “C” and “S” -shaped and with a characteristic small denticle at the both extremities (Fig 8G), 10-(13±2.2)-17.5 x 2 μm; sigmas II regular in shape 27.5-(31.7±2.7)-35 x 2 μm, not very common (Fig 9H); arcuate chelae 17.7-(29±6.7)-37.5 x 1-(2.3±0.4)-2.5 μm (Fig 9I).

Distribution: Known from Hawai’i only.

Remarks and discussion: In 1950a de Laubenfels [4] described the species *Damiriana hawaiiana* from Hawai’i. It was described as a brilliant red sponge, with a basal encrusting part from which little-finger branches arise. Its surface is smooth with oscules (about 6 mm) and pores grouped in cribrous areas. Spicules are tylostyles 170 x 4 μm, oxeas 200 x 8 μm and also 230 x 9 μm, with some shorter spicules; isochelas 27 μm and sigmas 13 μm. This last category of microscleres is described by the author with a sort of “inward pointing clad” that makes them resemble a “reduced chela”. In 2002a Van Soest re-examined the holotype of *D*. *hawaiiana* and considered it a probable junior synonym of *L*. *(W*.*) schmidi*. Also Van Soest detected a single category of small sigmas 13 μm long.

In the present material (collected in the same type locality around Coconut Island) two categories of sigmas were found. The larger one of about 30 μm is rather rare and the smaller one is characterized by small denticles at the extremities, clearly visible at the SEM observation. Also by optical microscope observation these denticles are visible and correspond to the clads reported by de Laubenfels [4]. These small denticles are not present in the holotype of *L*. *(W*.*) schmidti* [58] as shown by Van Soest [59] in the SEM pictures. Moreover also the shape of the isochelae is rather different especially in the shape of the frontal and lateral alae that in the Hawai’ian specimens are straight.

These data support enough evidence to reject the synonymy and considering the species of de Laubenfels as a valid taxon [4]. No barcoding data was available before the sequences we have submitted in the current study. For the ribosomal fragment our amplifications spanned from the partial end of 18S over the ITS-2. ABGD analyses showed 100% and 98.6% COI similarity with a *Lissodendoryx* sp. clone from Australia, and a Caribbean *L*. *stigmata* respectively, demonstrating poor species segregation with COI barcoding marker, or identification errors in the databases. Pairwise distances with other congener reference sequences (*L*. *isodictyalis*, *L*. *colombiensis*, *L*. *flabellata*) ranged 3.2–9.4%. Our ribosomal ITS fragment is at the moment the first record in GenBank for genus *Lissodendoryx*, so no informative genetic analysis can be discussed for this marker.

Family Crambeidae Lévi, 1963

*Monanchora clathrata* Carter, 1883

Fig 10

**Fig 10:**
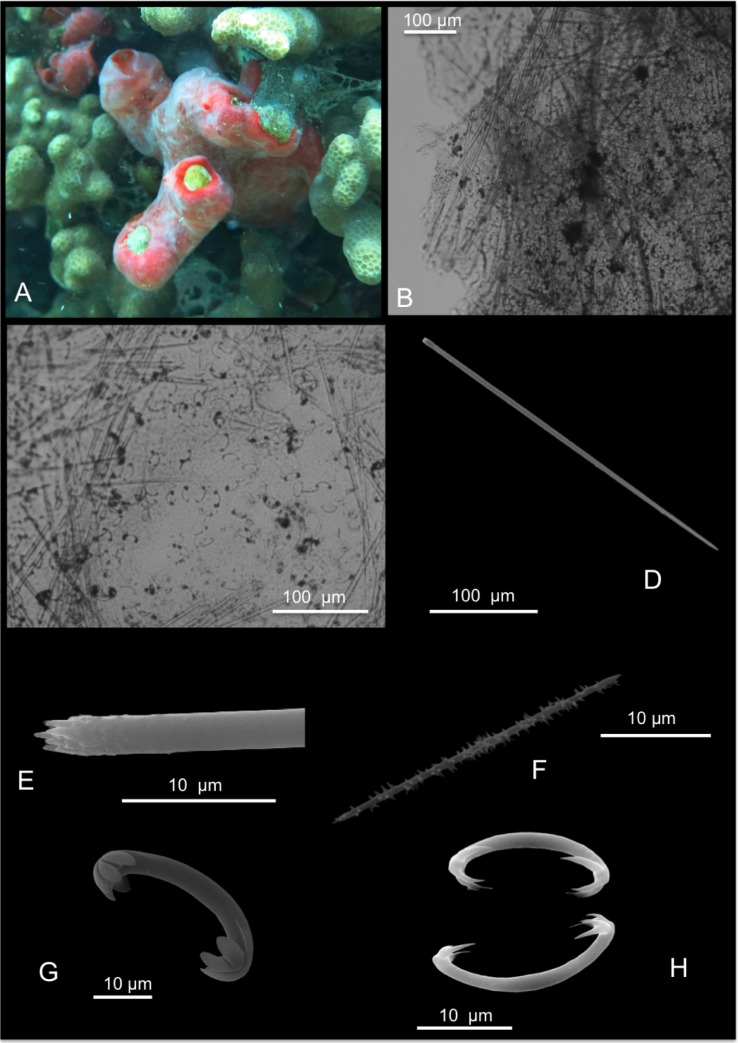
Monanchora clathrata. A specimen *in vivo;* B tracts of subtylostyles; C microscleres concentrated on the external membrane; D subtylostyle; E magnification of a subtylostyle tip ending with groups of small spines; F microspined microxea; G anchorate chela; H unguiferate chelae.

*Monanchora clathrata* Carter, 1883: 369 [60].

Material: SPO7, Coconut Is. Point Lab, 5 m. SPO38, Coconut Is. Point Lab, 5 m.

Description: Very thin sponge encrusting *Porites* branches; SPO38 is massively encrusting to lobate; on the surface ligher coloured canals converge to rounded, elevated oscules; the sponge is reddish, dull orange internally (Fig 10A); dark orange when preserved. The surface is smooth and translucent and it releases an intense foetid smell.

Skeleton: Due to the thinness of the sponge only vague tracts and brushes of spicules are recognizable (Fig 10B). Microscleres are concentrated on the external membrane (Fig 10C).

Spicules: Subtylostyles with slightly developed tyle and variable in thickness (Fig 10D). Often their tips end with a single or groups of small spines (Fig 10E). Microspined microxeas (Fig 10F). Anchorate chelae with 5 (Fig 10G). Reduced unguiferate chelae with 3–5 sharp-pointed teeth (Fig 10H). Measurements are shown in Table 6.

**Table 6:** Spicule measurements of *M*. *clathrata*.

	Subtylostyles (μm)	Microxeas (μm)	Anchorate chelea (μm)	Unguiferate chelae
SPO 7	265-(323.4±29.6)-390 x 2.5-(5.2±1.7)-7.5	25–31 x <1	27.5-(29.2±1.2)-30	26–36
SPO 38	235-(310.8±45.6)-375	24–30 x <1	28-(35±1.4)-37.5	20–25

Distribution: It was recorded in Western Australia [60] and Vietnam [61].

Remarks and discussion: The specimen from Hawai’i fits with the species *Monanchora clathrata* for its spicule shape and size (subtylostyles, considering a single category, 248–362 x 4–14 μm; microspined microxeas 36–57 x <0.5 μm; anchorate-unguiferate and reduced unguiferate chelae 26–36 μm [62]. In 2002b, Van Soest re-examined [62] the holotype and reported the presence of microxeas overlooked by the previous authors.

The main differences found in the present material respect to those previous descriptions relay in the reduced thickness of the subtylostyles and in growth shape of the sponge, which in our specimens is encrusting instead of massive, clathrous.

This species was already recorded in Hawai’i in massive shape (Pearl Harbour, O’ahu Island, Coll. S. L. Coles-H. Bolick–Bishop Museum–in 2007 and 2008, and identified by B. C.), but this is the first documented record of *M*. *clathrata* for Hawai’i. There were three previous COI sequences in NCBI database for this species, and now we provide the fourth one, plus the first ribosomal nucleotide data record. In comparison with three reference *M*. *clathrata* sequences from Indonesia our specimen had 0%, 0.1% and 0.4% divergence. Genetic distances displayed lower values than standard species delimitation values with *M*. *quadrangulata* from the Red Sea (0.5%) and the Caribbean *M*. *arbuscula* (2.1%), suggesting poor resolution of COI marker within this genus. From a morphological point of view, these two species also differ from *M*. *clathrata* in very subtile features regarding the spicules dimensions (in the case of *M*. *arbuscula* [63]); and shape (with spicules consisting in very thin subtylostyles in *M*. *quadrangulata* [64]). There were no GenBank references for *Monanchora* ITS ribosomal regions to compare with our data, but the closest matching sequence (97.5% similarity) was from a Mediterranean *Crambe crambe* (Catalonia).

Family Iotrochotidae Dendy, 1922

*Iotrochota baculifera* Ridley, 1884

Fig 11

**Fig 11:**
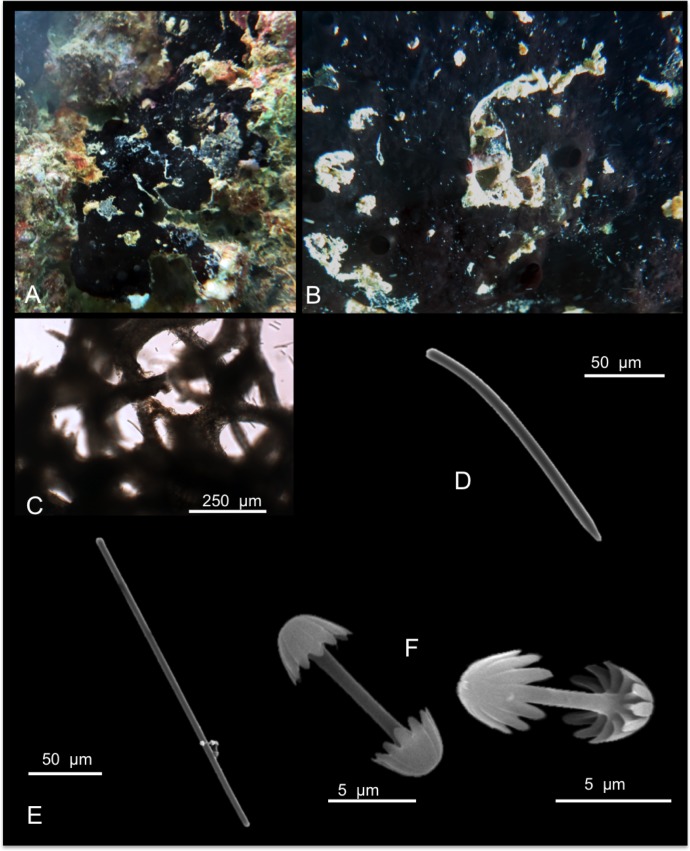
Iotrochota baculifera. A, B alive specimens. C choanosomal skeleton; D stout style; E anisostrongyle; F birotulas.

*Iotrochota baculifera* Ridley, 1884 [58]: 435; *Hiattrochota protea* Laubenfels, 1950a [4]: 20; *Iotrochota protea* de Laubenfels, 1950a: 20 [4].

Material: SPO13, Coconut Is. Point Lab, 3 m.

Description: Massive to massively encrusting sponge. The colour is brilliant black to very dark purple (Fig 11A and 11B); the colour persists in alcohol. The sponge is slimy and exudes dark purple slime in large quantity when collected. The surface *in situ* is smooth with wide, elevated scattered oscules; the surface became irregular in alcohol-preserved specimens. Specimens were covered by microconules created by the choanosomal tracts running towards the surface. Microscopic observations revealed numerous scattered cribrous areas. The sponge is cavernous and has a spongy elastic consistence, but it is difficult to tear.

Skeleton: The surface of the sponge is covered by a thin dermal membrane that collapses after preservation, making the surface microconulose. In the choanosome the skeleton consists of a regular reticulation of ovoid meshes, about 250–650 μm wide, and plurispicular tracts of styles about 90–200 μm in diameter (Fig 11C). Spongin is not evident.

Spicules: Stout styles, with triangular tips (Fig 11D); these are curved in proximity of the apical rounded extremity; they measure 145-(160±7.4)-170 x 5-(7.5±1.2)-8.7 μm. Anisostrongyles (Fig 11E) in general straight, they measure 205-(220.9±8.6)-230 x 2.5-(4±1)-5 μm. Microscleres are birotulas, about 12 μm long (Fig 11F).

Distribution: This species was recorded in O’ahu and in other islands of the Hawai’ian Archipelago by de Laubenfels [4]. For the general distribution details see below.

Remarks and discussion: In the Indo-Pacific area there are eight related species reported [27]: *Iotrochota acerata* Dendy, 1896 [65] and *I*. *coccinea* (Carter, 1886) from Australia [66]; *I*. *iota* (de Laubenfels, 1954b) and *I*. *membranosa* (Esper, 1794) were described for the West Pacific ocean [67,68]; *I*. *nigra* (Baer, 1906) is known from Tanzania [69]; *I*. *pella* de Laubenfels, 1954b is known from the Marshall Islands [67]; *I*. *purpurea* (Bowerbank, 1875) was recorded in Indonesia and in the Indian ocean [70]; *Iotrochota protea* (de Laubenfels, 1950a) from Hawai’i [4]. Finally *I*. *baculifera* Ridley, 1884 is widely diffused in the Indo-Pacific area [58].

*I*. *acerata* has styles, smooth oxeas and strongyles about 200 μm long as megascleres [65]. *I*. *coccinea* and *I*. *nigra* have only styles as spicules [66,69]; *I*. *iota* is encrusting and has styles 125 x 4 μm and birotules 13 μm long [67]. *I*. *pella* is an encrusting, black sponge with only strongyles as megascleres [67]; *I*. *purpurea* has two types of styles [670]. *I*. *membranosa* is not clearly described by the author [68].

De Laubenfels [4] described *I*. *protea* as a massive, black sponge with smooth surface and pores concentrated, (probably) in cribrous areas. In the choanosome there are “few vague spicular tracts about 50 μm in diameter” and “the skeleton [..] approaches the isodictyal condition”; the endosome is microcavernous. Spicules are smooth strongyles, styles and birotules (amphidiscs). Their measurements are reported in Table 7.

**Table 7:** Comparison of spicule measurements (μm) in specimens of *I*. *baculifera* and *I*. *protea*.

	Styles (μm)	Strongyles (μm)	Birotules (μm)	References
*I*. *baculifera*	200 x 9.5–12.7	220–280 x 6.3	16	Ridley, 1884
*I*. *baculifera*	170–210 x 6–11.5	250–290 x 5–6.3	14.5	Pulitzer-Finali, 1993
*I*. *baculifera*	125–180 x 5.5–7.5	225–255 x 3.5–5	13–16.5	Bergquist, 1965
*I*. *protea*	135–180 x 7–10	140–205 x 3–6	12–13 (up to 15 in others specimens)	Laubenfels, 1950a

*I*. *baculifera* is black and erect, “formed of subcylindrical lobes, terminating bluntly” […] “Surface chiefly rough, owing to the projection from it, at intervals of 5 to 1 millim., of blunt meandering ridges or conical blunt processes”. The skeleton consists of wide meshes 400–600 μm wide, made by 12–15 spicules (styles). Spicules are smooth, curved styles, straight with rounded ends strongyles and birotules. Measurements are in Table 7. The specimen from Coconut Island, here described, fits with the species *I*. *baculifera* Ridley [58].

*I*. *protea* appears very similar to *I*. *baculifera* for its shape, colour, surface features, tessiture and spicules features (see Table 7). Bergquist [71] pointed that these two species are probably synonyms and the “only features which distinguish them are the massive form and the absence of a well-defined skeleton of spicules tracts in *I*. *baculifera*”. Berguist [71] recorded in Palau encrusting specimens of *I*. *baculifera*, but actually Ridley [58] described the sponge as erect and with subcylindrical lobes.

Van Soest [72] re-examined the type species of *I*. *protea* (assigned to the genus *Hiattrochota*) and allocated it to the genus *Iotrochota* Ridley, 1884, considering that there were no elements to distinguish it from *Iotrochota*. In the same paper, in the diagnosis of the genus, the choanosomal skeleton of *Iotrochota* is defined as a regular reticulation of multispicular tracts [72]. As consequence, also in *I*. *protea* there is a regular reticulation and there are no valid reasons to consider *I*. *protea* distinct from *I*. *baculifera*; as consequence, the latter should be considered as the older synonym. There were three available COI sequences of *I*. *baculifera* and we now submit another record. Our specimen showed high percentage of similarity with the available conspecific reference sequences (98% and 97.6%). But also, p distance analyses revealed low values for species delimitation against conspecifics like *I*. *acerata* (2.3%) and *I*. *coccicea* (2.4%) from Australia. Congruent distances were found instead with *I*. *birotulata* (3.9%). Again, the COI marker seems to not discriminate some species within the genus *Iotrochota* when applying the standard 2.5% threshold. For the ribosomal region, there was reference of the 28S partial gene, but no available conspecific or congeneric references were available of ITSs markers. Thus a relevant genetic distance analysis could not be done. Here we provide the first 18S-ITS1-5.8S-ITS2-28S fragment for this species.

Family Mycalidae Lundbeck, 1905

*Mycale (Mycale) grandis* Gray, 1867

Fig 12

**Fig 12:**
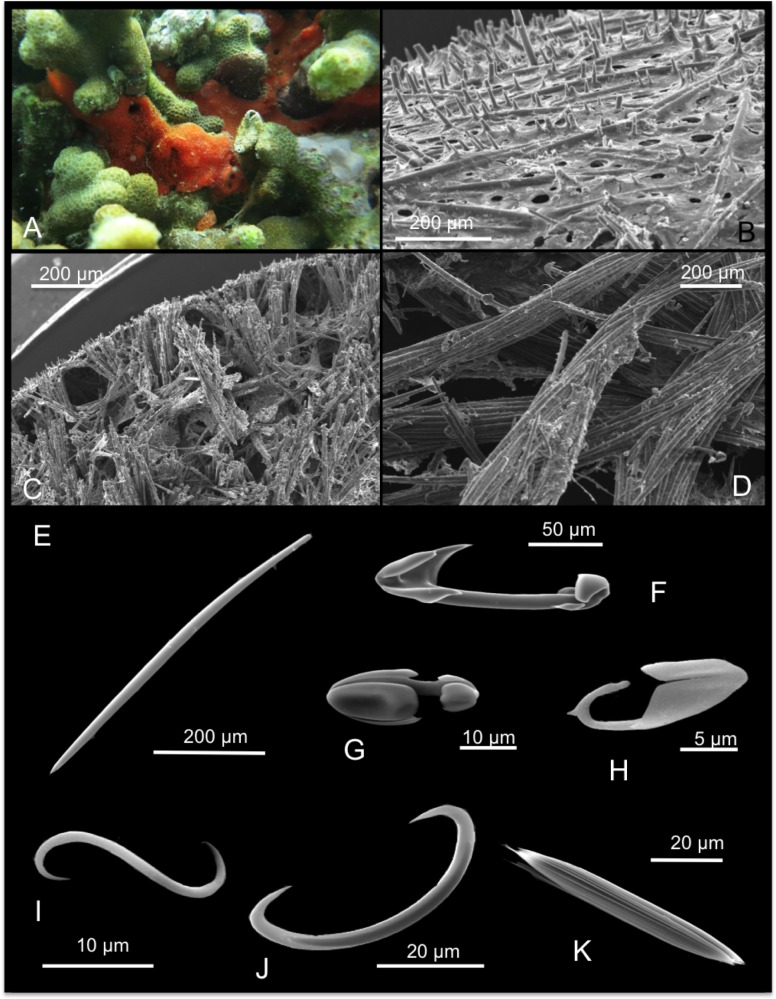
Mycale (Mycale) grandis. A alive specimen; B ectosomal skeleton; C choanosomal skeleton; D fibres echinated by the larger anisochelae; E mycalostyle; F anisochela I; G anisochela II; H anisochela III; I sigma I; J sigma II; K raphides in trichodragma.

*Mycale grandis* Gray, 1867: 533 [73]; Hentshel, 1912: 337 [39].

Material: SPO4, Coconut Is. Point Lab, 3 m; SPO19, Lagoon Floating Deck, 0.2 m;

SPO43, reef 23, 2.5m; SPO44, reef 24, 3.5 m.

Description: Massively encrusting sponge that can adopt remarkable massive shapes covering corals and other substrates. The colour is vivid red, or orange (Fig 12A), and turns whitish in alcohol. *In situ* it has wide, prominent oscules, in which exhalant evident cannels converge; the surface is irregular, not optically smooth. The sponge is soft and compressible.

Skeleton: the ectosomal skeleton is made of intercrossing, tangential tracts of mycalostyles and microscleres (Fig 12B). The choanosomal skeleton is plumoreticulate, with ascending fibres of mycalostyles (100–300 μm) diverging towards the surface (Fig 12C). Sponginis scarce. The fibres are often echinated by the larger anisochelae (anisochelae I) (Fig 12D). Sigmas are numerous in the choanosome.

Spicules: Mycalostyles straight or slightly curved (Fig 12E); anisochelae I (Fig 12F), long and with straight shafts; anisochelae II palmate (Fig 12G); anisochelae III palmate with a basal spur (Fig 12H); sigmas I “C” and “S” shaped (Fig 12I); sigmas II “C” and “S” shaped (Fig 12J); raphides in trichodragmas (Fig 12K).

Measurements in Table 8.

**Table 8:** Spicule measurements of *Mycale (M*.*) grandis*.

	Mycalostyles (μm)	Anisochelae I (μm)	Anisochelae II (μm)	Anisochelae III (μm)	Sigmas I (μm)	Sigmas II (μm)	Raphides (μm)
SPO 4	510-(583.7±41.8)-670 x 12.5-(15.9±2.3)-20	120-(135.1±6.8)-145	25-(28.7±2.7)-35	15-(17.2±2)-20	17.5-(19±1.5)-20	37.5-(47.2±5.9)-55	35.5-(52.3± 9.1)-71.2
SPO 19	540-(577.5±22.6)-610 x 12.5-(15.7±2)-17.5	120-(127±6.7)-137.5	30-(33±3)-37	20	17.5-(20.4±3)-25	50-(52±1.8)-55	50-(65.8±9)-85
SPO 43	480-(527±26.9)-570 x 7.5-(13.5±3.2)-17.5	115-(128±7.5)-140	30-(34.1±3.7)-40	17.5-(24±5)-27	15-(16.8±1.4)-19	45-(50±4.4)-55	32-(54±14)-60.5
SPO 44	520-(575±38.4)x10-(13.3±2.1)-16.2	120-(131.6±7.5)-140	25-(32.1 ± 3.1)-36	15-(16.1±1.1)-19	17-(18.9±1.2)-20	35-(38.9±3.4)-45	35-(45±19.6)-80

Distribution: Widely diffused in the Indo Pacific Ocean [27]; the native range of *Mycale grandis* (Orange Keyhole sponge or previous cited as *Mycale armata*, Thiele) is Australia (GBR), Torres Straits and the Indo-Malay region.

Remarks and discussion: The fouling habit and invasive capability of this species have permitted its establishment in the main Hawai’ian Islands: O’ahu–Pearl Harbour, Honolulu Harbour, Keehi Lagoon, Barber’s Point Harbour, and Kane’ohe Bay; and Maui–Kahului Harbour. Here *M*. *(M) grandis* is restricted to shallow-water fouling communities of the major harbours on O’ahu or associated disturbed habitats. In Kane’ohe Bay, *M*. *(M) grandis* inhabits on southeastern patch reefs, and has its maximal coverage in the vicinities of Coconut Island ([2,74]; Authors’ unpublished observations).

*Mycale (M) grandis* is considered an unintentionally introduced species to Hawai’i due to its sudden appearance in the islands. It was discovered in Pearl Harbour in 1996 [75]. Its notable abundance in Kane’ohe Bay and bright orange coloration makes it quite unlikely that this species could have been overlooked by de Laubenfels [4,6,8,37] and Bergquist [9,10]. The ecological impact of *M*. *(M) grandis* is still understudied, but it seems to be invasive displacing native sponge and coral species. Yearly, the sponge cover in this species increased by a mean of 13%, while coral cover decreased by 16.3% [2]. The existing nucleotide data for this sponge include fragments from the large and small ribosomal subunit genes (18S and 28S). Here we afforded the first two ITS ribosomal sequences spanning from the end of 18S to the beginning of 28S, which were 100% similar among each other after gaps curation (on GBlocks), and also matching with *M*. *(Zygomycale) parishii* (from this study) and a *Mycale* sp. from China. Our sequences were instead clearly different from other two *Mycale* sp. (13.1% abd 50.9% dissimilarity), and a *Mycale fibrexilis* (25.8% p distance). We also submitted the first 5’ Folmer partition of the COI marker without the Erpenbeck’s ‘I3-M11’ extension, which could not be achieved after many efforts. There was clear divergence with other congeneric *Mycale* COI references, with p distances revealing values of 7.9% against *M*. *laxissima* and *M*. *mirabilis*, 12.4% with a *M*. *fibrexilis*, and 3.1% with *M*. *(Zygomycale) parishii*.

*Mycale (Zygomycale) parishii* (Bowerbank, 1875)

Fig 13

**Fig 13:**
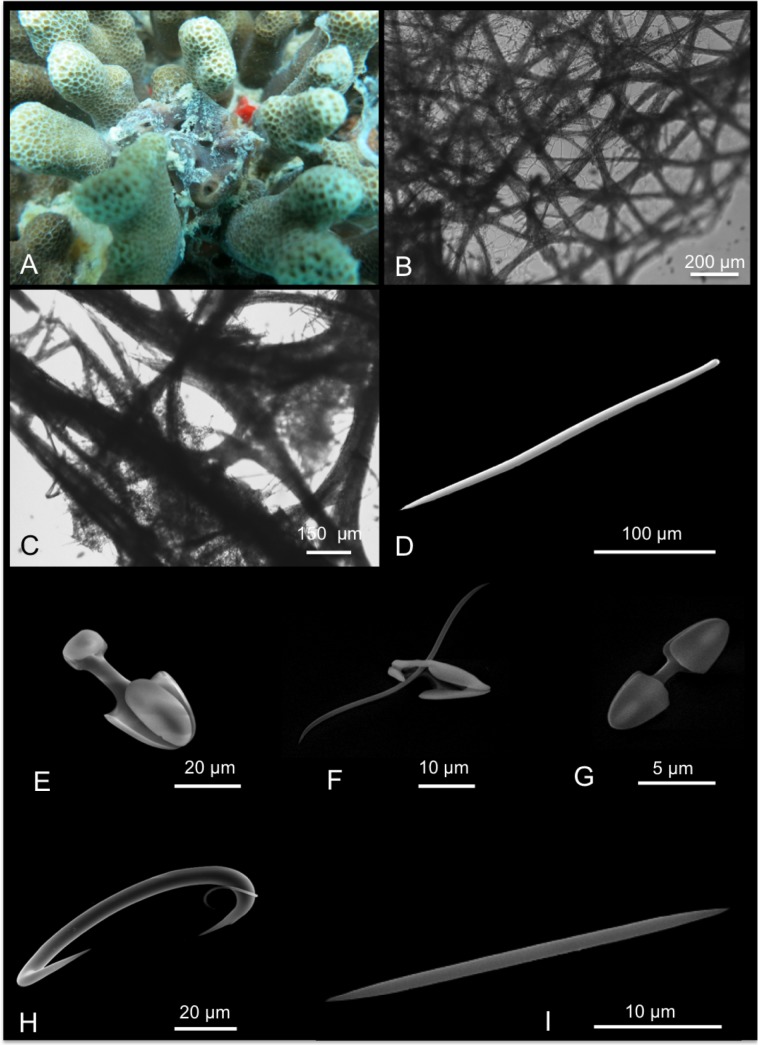
Mycale (Zygomycale) parishii. A alive specimen; B ectosomal skeleton; C choanosomal skeleton; D mycalostyle; E palmate anisochela I; F palmate anisochela II and toxa; G isochela; H sigma I and sigma II; I microxea.

*Raphiodesma parishii* Bowerbank, 1875: 283 [70]; Van Soest & Hajdu 2002d: 687 [76].

Material: SPO33, Reef 25, 3 m

Description: Massive sponge with prominent oscules and growing among corals (Fig 13A). The colour is maroon, and becomes whitish in alcohol. The sponge is soft and fragile.

Skeleton: In the ectosome tangential tracts of mycalostyles intercross creating regular triangular meshes (Fig 13B). The choanosomal skeleton is plumose made by multispicular tracts of mycalostyles (Fig 13C).

Spicules: Mycalostyles are often slightly flexuous or curved (Fig 13D), they measure 250-(300.6±25.2)-335 x 5-(7.5±1.8)-4 μm; palmate anisochelae I and II similar in shape (Fig 13E and 13F), respectively they measure 45-(50±3.1)-52.5 and 17.5-(20±2.9)-22.5 μm; isochelae, very small and thin (Fig 13G), they measure 4-(4.3±0.5)-5 μm; toxas (Fig 13F) 37.5-(59.6±21.4)-95 μm; sigmas I 65-(77.9±6.7)-85 μm and sigmas II 25-(29±3.3)-32.5 μm (Fig 13H), microxeas (Fig 13I) 30-(36.9±5.2)-42.5 μm.

Distribution: Widely present in the Indo-Pacific (see [12]).

In Hawai’i this species was first collected from Kane’ohe Bay at Coconut island by de Laubenfels [4], who described it as one of the commonest species at Coconut island, but rare or absent elsewhere in the bay. He also further noted its particularly abundance on vessels hulls in Pearl Harbour and Bergquist [9] again recorded it from the floating docks on Coconut Island, O’ahu and considered it introduced.

At present, O’ahu (Pearl Harbour, Honolulu Harbour, Keehi Lagoon, Barber’s Point, and Kane’ohe Bay) and Maui (Kahului Harbour) are the main Hawai’ian locations for this sponge species, where it is mainly restricted to shallow-water fouling communities of the major harbours and associated disturbed habitats [4,6,8,9,10,37]. This species displayed difficulties to amplify its DNA, in this work we report the first barcoding data of the COI, but only for the standard Folmer partition (491bp), and also ribosomal nucleotide sequence extending from the end of the 18S to the ITS-2 (338bp). Due to the relatively short span of the sequences obtained respect from those from the rest of the species, the distance analyses are not as reliable. Some values display suspiciously high similarities with non-congeneric sponges. The p distances with the other reference *Mycale* and the *M*. *grandis* from the dataset are constantly ~3.1%.

Family Tedanidae Ridley & Dendy, 1886

*Tedania (Tedania) ignis* (Duchassaing & Michelotti, 1864)

Fig 14

**Fig 14:**
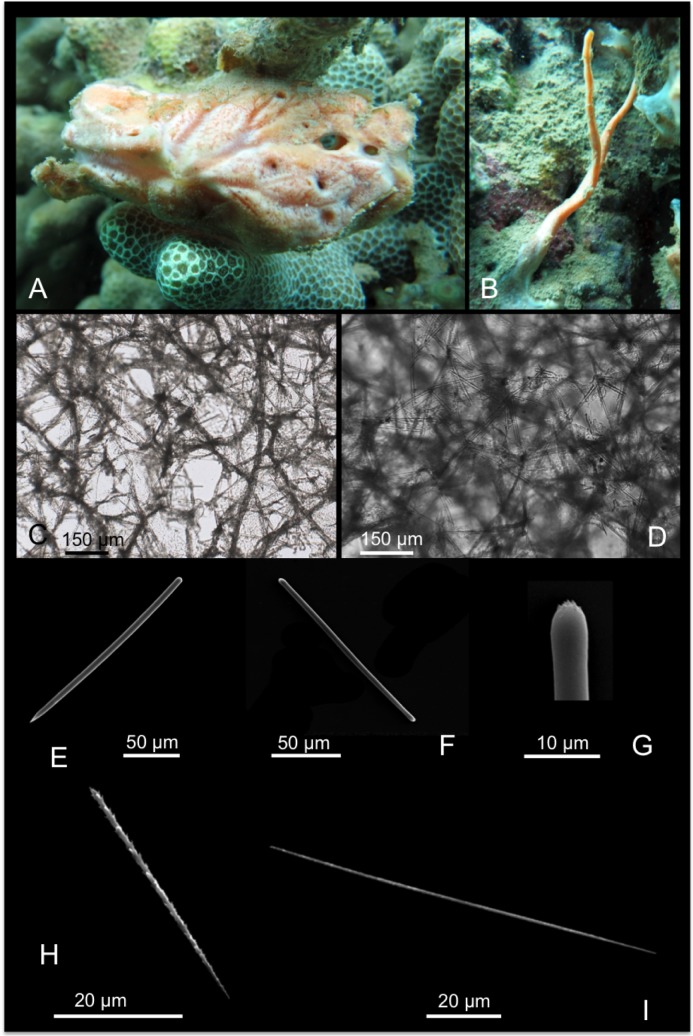
Tedania (Tedania) ignis. A alive specimen; B alive specimen with flattened projections; C choanosomal skeleton; D irregular isotropic reticulation of the choanosomal skeleton; E style; F tornote; G magnification of the microspined tip; H onychaete I; I onychaete II.

*Thalysias ignis* Duchassaing & Michelotti, 1864: 83; de Laubenfels, 1950a: 21 [4].

Material: SPO 6, Coconut Is. Point Lab, 3 m; SPO 36, Reef 44, 4 m.

Description: Massive or massively encrusting sponge, frequently observed growing among *Porites* spp. branches. Fresh material varies from pink salmon or skin cream to orange (Fig 14A); beige in alcohol; the surface is irregular with grooves covered by a translucent membrane (Fig 14A). The consistence is soft to slimy. Specimens can exhibit flattened fistule-like projections branching from the basal portion were observed (Fig 14B).

Skeleton: In the ectosome tylotes and onychaetes protrude from the surface making it hispid; paucispicular tracts of tylotes are tangentially disposed. In the choanosome styles make an irregular isotropic reticulation (Fig 14C and 14D).

Spicules: Styles frequently curved (Fig 14E); tornotes with rounded and microspined points (Fig 14F and 14G); onychaetes I have clear asymmetrical tips and slightly spread, long spines (Fig 14H); onychaetes II finely spined with symmetrical tips (Fig 14I). Spicule measurements are shown in Table 9.

**Table 9:** Spicule measurements of *Tedania (T*.*) ignis*.

	Styles (μm)	Tornotes (μm)	Onychaetes I (μm)	Onychaetes II (μm)
SPO 6	180-(192.5±9.4)-202.5 x 5-(6±1.5)-7.5	160-(181.8±9.2)-192.5 x 2.5-(4±1.2)-5	125-(136±13.4)-180	55-(59±2)-62
SPO 36	190-(208±13.7)-235 x 6.2-(7±0.6)-7.5	205-(208.3±13)-210 x 3.7-(4.1±0.6)-5)	95-(114.2±14.2)-135	60-(62±2)-65

Distribution: Bermuda, Brazil, Caribbean, Panama (de Laubenfels [37]), and Palau (de Laubenfels [67]).

Remarks and discussion: *Tedania ignis* is very common throughout the shallow waters of Hawai’i. It was reported from K-Bay (O’ahu) and in the Island of Hawai’i near Hilo, Kaalualu, and Honaunau [4]. Hiatt also collected this sponge at Halape (Hawai’i) for de Laubenfels [4] inventory. The species has been considered an introduction to Hawai’i since 1950 (see [4,9,11]). Several sequences are available in NCBI database for the standard Folmer partition of COI, and also for the large and small ribosomal subunit genes (18S and 28S). Here we contribute with two additional records: the COI partial fragment with the Erpenbeck’s ‘I3-M11’ extension, and the nuclear ribosomal fragment covering 18S-ITS1-5.8S-ITS2-28S. The COI region revealed 100% match between samples SPO6 and SPO36, and similarities ranging 1.4% to 2.1% with conspecific reference records from Panamà, Belize and Vietnam. Species delimitation was not as clear against a *T*. *klausi* from Belize (1.9% divergence), while p distances yielded values rounding 2.7% against *T*. *massa*, *T*. *oxeata* and *T*. *trirhaphis* from New Zealand. For the ITS markers there were no congeneric reference sequences, whereas the divergence values displayed 2.1–2.5% between SPO6, SPO36 and a reference T. *ignis* from Bermuda. These results support the alloctonous origin of the hawai’ian specimens.

Order Biemnida

Family Biemnidae Hentschel, 1923

*Biemna fistulosa* (Topsent, 1897)

Fig 15

**Fig 15:**
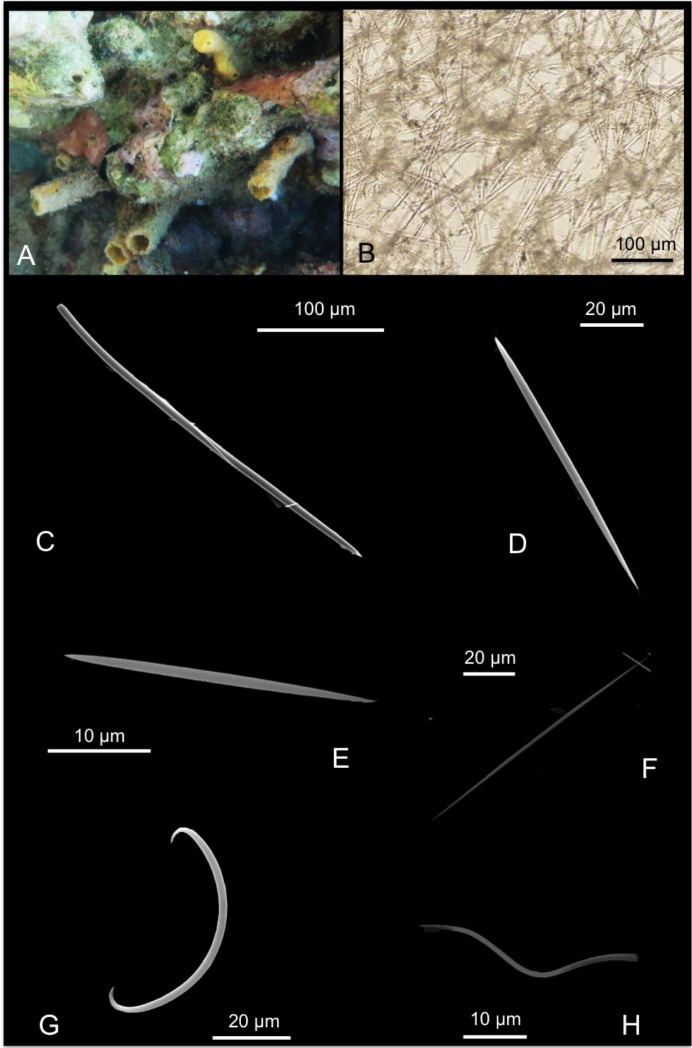
Biemna fistulosa. A alive specimen; B choanosomal skeleton; C style; D microxea I; E microxea II; F raphide; G sigma; H comma.

*Desmacella peachi* var. *fistulosa* Topsent, 1897: p. 462 [50].

Material: SPO 1, Coconut Is. Point Lab, 4 m. SPO 3, Coconut Is. Point Lab, 5 m.

Description: Sponge with fistules often coalescent and insinuating among corals or growing inside small crevices (Fig 15A). The sponge is yellow, sometimes darker brown-reddish in colour (Fig 15A). The examined samples consist of elongated, fragile fistules of variable dimension, 5–15 cm long and ~1 cm wide.

Skeleton: The choanosomal skeleton is plumoreticulate, and built mainly by styles in multispicular tracts (Fig 15B).

Spicules: Styles slightly curved (Fig 15C); microxeas I with smooth or slightly microspined tips (Fig 15D); microxeas II with one microspined tip (Fig 15E); raphides (Fig 15F); sigmas (Fig 15G); commas (Fig 15H). See Table 10 for measurements.

**Table 10:** Spicule measurements of *Biemna fistulosa* (μm).

	Styles (μm)	Microxeas I (μm)	Microxeas II (μm)	Raphides (μm)	Sigmas (μm)	Commas
SPO 1	275-(302.5±15.6)-330 x 5-(7.3±1.3)-8.7	87.5 (100.8±7)-115 x <2	25-(29.7±3)-35 x 2	10470-(104±11.5)-125 x <2	13-(45±9.4)-32 x 2	about 30
SPO 3	300-(306.7±10.4)-310 x 7- (8.6±0.9)-10	80 (98±8)-102.5 x <2	22.5-(27.3±2.7)-32.5 x 2	87.5-(108.4±7.4)-120 x <2	17.5-(35.7±10.7)-47.5 x 2	about 30

Distribution: Ambon, Hong Kong, East Africa and Hawai’i (see [12]). In Hawai’i this sponge was recorded in O’ahu Island, Hawai’i Kai and Pearl Harbour [75,12].

Remarks and discussion: This species has a rich spicular feature very characteristic; our specimens of *Biemna fistulosa* fit with the description of the species made by Topsent [50]. *B*. *fistulosa* was recently recorded in Hawai’i growing on *Carijoa riisei* [12]. There are two barcoding records of *B*. *fistulosa* for the standard Folmer COI partition, and also one including the Erpenbeck’s ‘I3-M11’ extension. In this study we submitted a standard COI partition with the recommended extension matching with 100% similarity with all the conspecific references. Pairwise distances showed good species delimitation with available conspecifics: *B*. *ehrenbergi* (4%), *B*. *variantia* (5.5%) and *B*. *saucia* (6%). This sponge also has reference sequences for the 18S and 28S genes, but not for the ribosomal ITS. Thus, we could not contrast for species delimitation on this marker with our 18S-ITS1-5.8S-ITS2-28S fragment.

Order Axinellida

Family Raspailiidae Nardo, 1833

*Echinodictyum asperum* Ridley & Dendy, 1886

Fig 16

**Fig 16:**
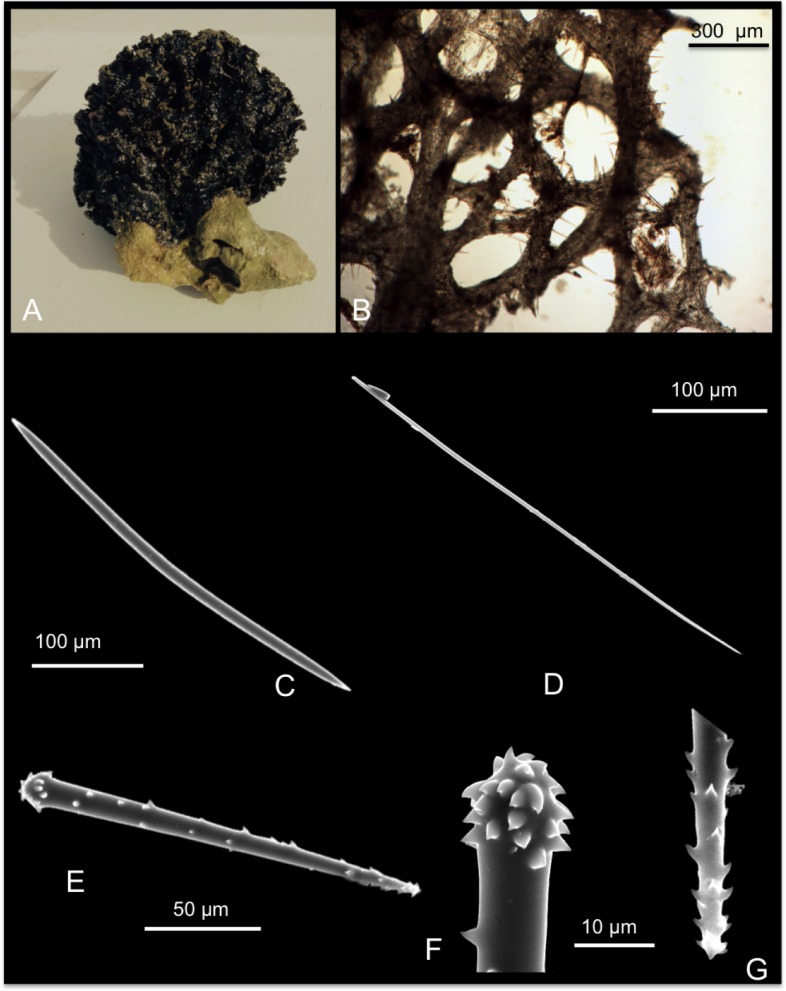
Echinodictyum asperum. A fresh collected specimen; B choanosomal skeleton; C oxea; D straight style; E club-shaped acanthostyle; F magnification of the acanthostyle head; G magnification of the acanthostyle tip.

*Echinodictyum asperum* Ridley & Dendy, 1886: 477 [47]; Hooper, 1991: 1353 [77].

Material: SPO 41, Reef 1 HIMB, 0.5 m.

Description: Spherical sponge of about 15–25 cm in diameter growing on dead coral rubbles (Fig 16A) and sandy shallows. The sponge is cavernous and consists in flattened, arborescent branches. The coloration is black in *vivo* as well as when preserved (Fig 15A). The examined specimen is a portion of about 6 cm long. The surface is irregular and conulose. The texture is firm and difficult to tear.

Skeleton: In the ectosome and choanosome oxeas are organized in compact fibres of about 100–300 μm in diameter. Acanthostyles strongly echinate the fibres both in the choanosome (Fig 16B) and ectosome. Meshes are about 300–500 μm in diameter and can reach up to 1300 μm.

Spicules: Slightly curved or straight oxeas with mucronate, acerate often rounded tips 210-(290±39.2)-420 x 10-(11.8±2.2)-15 μm (Fig 16C); numerous very thin oxeas are common; thin, straight styles 250-(362±87.9)-500 x 2.5–4 μm (Fig 16D). Echinating, club-shaped acanthostyles 105-(136±12.4)-160 x 20 μm (Fig 16E); spines are concentrated on the head and on the tip of the spicules (Fig 16F). The tips end with an apical spine (Fig 16G), not always present; often the extremity appears truncate at the optical microscope observation.

Distribution: Present widely in the Indo-Pacific, distributed from the Arabian Gulf through the Indo-Pacific, including the western coast of Australia, the Palau marine lakes, Guam, Likiep, Pohnpei, northern and southern Papua New Guinea and Zanzibar, and Hawai’i.

Remarks and discussion: Our specimen fitted with the description of the species made by Ridley & Dendy [47] and with the accurate re-description by Hooper [77] based on new records from Australia. It was documented in Pearl Harbour [78]. This sponge could have been overlooked in previous reports since it can be found covered with sediment. There were no available nucleotide barcoding records of this species previous to this work. Here we afford the partial mitochondrial COI sequence and the ribosomal fragment. The COI showed a reliable species delimitation of 3.1%, 4.2% to 5.1% divergence with congenerics such as *E*. *cancellatum*, *E*. *clathroides* and *E*. *mesemterinum* respectively. Similar analyses were not posssibe on the ITS ribosomal marker, due to the lack of congeneric entries.

Order Haplosclerida

Family Callyspongiidae de Laubenfels, 1936

*Callyspongia (Cladochalina) diffusa* (Ridley, 1884)

Fig 17

**Fig 17:**
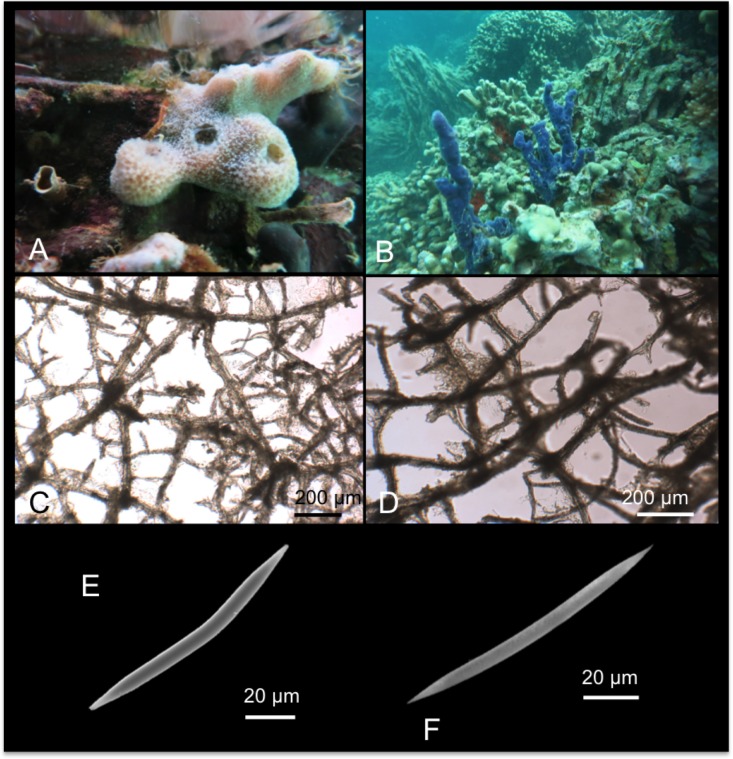
Callyspongia (Cladochalina) diffusa. A specimen SPO16 *in situ*; B specimen SPO 8 *in situ*; C ectosomal skeleton; D choanosomal skeleton; E, F oxeas.

*Cladochalina diffusa* Ridley, 1884: 183 [58].

Material: SPO 8, Coconut Is., Point Lab, 5 m; SPO 16, Coconut Is., Lagoon Floating Deck, 0.3 m; SPO 20, Coconut Is., Lagoon Floating Deck, 0.2 m.

Other material: NHM 82.10.17.57 Holotype.

Description: Massive, digitate sponge (Fig 17A) with short flat branches (SPO 16 and SPO 20); oscules are evident and about 2–6 mm wide in the preserved specimens; they are on the top of the tubular branches, or irregularly distributed. Specimens SPO 16 (Fig 17A) and SPO 20 are light beige, darker in alcohol; specimen SPO 8 is erected, arborescent with cylindrical branches; the colour of this specimen is light blue, cerulean (Fig 17B), beige in alcohol. The superficial web is visible at the naked eye, so that the surface is glabrous especially in the living specimens. In SPO 20 the surface is smooth, while in Spo 8 it is shaggier. The texture is compressible and elastic.

Skeleton: The ectosomal skeleton (Fig 17C) is made of subquadrangular meshes about 60–300 μm wide made of primary and secondary, plurispicular fibres. Primary and secondary fibres are about 30–80 μm in diameter; paucispicular (3–5 spicules), tertiary fibres are about 10–20 μm in diameter. Choanosomal skeleton more disarranged with clearly detectable plurispicular, primary fibres of about 100–200 μm in diameter and secondary of about 40 μm in diameter (Fig 17D).

Spicules: Slightly curved and pointed oxeas (Fig 17E and 17F). Measurements can be consulted in Table 11.

**Table 11:** Spicule measurements of *Callyspongia (C*.*) diffusa*.

	Oxeas (μm)
SPO 8	77.5-(86±4.2)-90 x 2.5-(4.7±1.4)- 7.5
SPO 16	105-(116.5±7.7)-135 x 5-(6.1±41)-7.5
SPO 20	95-(110.2±48.4)-120 x 3.7-(6.2±1.7)-7.5
Holotype NHM 82.10.17.57	87-(101.2±7.4)-115 x 2.5-(6.2±1.6)-7.5

Distribution: Widely distributed in the Indo-Pacific Ocean ([27]). It was first described as *Cladochalina diffusa* by Ridley [58] from the Indian Ocean. Subsequently it was recorded as *Cladochalina elegans* by Lendenfeld [79] from South Australia, as *Chatina pulvinatus* by Lindgren [80] from the Malay region, and as *Ceraochalina retiarmata* by Dendy [81] from India. It was reported in Hawa’i for the first time by de Laubenfels [4].

Remarks and discussion: The specimen SPO 8 is morphologically, slightly different from the other two specimens analyzed (SPO 16 and SPO 20) for its erected, arborescent shape and it is blue colour. Its spicules are slightly shorter (Table 11), also respect to the original type description (110 x 63 μm, [58]).

The specimens collected in Hawai’i of *C*. *(C*.*) diffusa* fit with the description of the species; the comparison with the type material allowed us to confirm the identification.

Ridley [58] described the species as suberect, branching, displaying a surface even or echinated by few sharp vertical projections, and firm in texture, but compressible and elastic. The main skeleton was described as rectangular; meshes about 400 μm; primary fibers about 100–140 μm; secondary fibres 70–100 μm. Dermal skeleton forming subquadrate meshes 180–360 μm made of fibres 25–100 μm tick. Spicules were 110 by 6.3 μm. The colour of those specimens was also reported as violet and brown [82].

*Callyspongia diffusa* is moderately common throughout the Hawai’ian Islands. It was reported by de Laubenfels [4] from Coconut Island and Waialua Bay in 1947, Kailua (O’ahu) in 1948, and also from Halape (Hawai’i) in 1945 (Hiatt’s collection). There were no prior reference sequences designated to this species before the present study. Here we submitted three COI and three ribosomal partial marker nucleotide entries, one per specimen voucher. Sequences from SPO16 and SPO20 and *Callyspongia* sp. SPO30 (see also next species description) were 100% maching for the COI partition, and were 99.9% similar to SPO8 (*C*. *diffusa* purple morph). All four specimens were also 100% similar to a reference entrie corresponding to a *Callyspongia* sp. sponge from Oman, which could potentially correspond to a *C*. *diffusa*. Instead, when contrasted with other congeneric sequences we found reliable species delimitations, with divergence percentages recording ~3.2% against *C*. *siphonella*, ~4.4% with *C*. *fallax* and *C*. *ramosa*, 7.8% against *C*. *(Toxochalina)* cf. *pseudotoxa* (SPO34 form this study), ~8.7% with *C*. *plicifera*, ~12.4% against *C*. *vaginalis* and ~14.5% with *C*. *armigera*. The ribosomal marker for the 18S-ITS1-5.8S-ITS2-28S fragment also yielded 100% match for samples SPO8, SPO16, SPO20 (all identified as *C*. *diffusa*) and SPO30 (*Callyspongia* sp.), as well as again with an Arabic *Callyspongia* sp. (Oman). Skeletal organitation and spicule size and shape are similar in these all four specimens (SPO 8, SPO 16, SPO 20 ans SPO 30). SPO 30 though, is notoriously different from the others in the external morphology being massive and in displaying a strongly conulose surface. Considering that we have analysed only one specimen with these external morphological characteristics and that among haplosclerids, characters are simple and often not diagnostic, we prefer to maintain the specimen SPO 30 as a separate taxonomic entity. The only available conspecific sequence to calculate p distance was that of *Callyspongia (Toxochalina)* cf. *pseudotoxa* SPO34 from the present study, which was 13.1% dissimilar.

*Callyspongia (Cladochalina)* sp.

Fig 2C

Material: SPO 30, reef 22, 5 m.

Description: Massive, sponge, red-brown to grey translucent sponge with shimmering fibres. It has a strong conulose surface and is very spongy to touch and difficult to tear. Beige in alcohol.

Ectosomal and choanosomal skeleton are quite irregular. Primary, secondary and tertiary plurispicular fibres (up to 100) create polygonal meshes. Spongin is evident.

Spicules: oxeas of about 90–110 x 5 μm.

Remarks and discussion: The sequences here submitted for the COI marker (standard 5’ Folmer partition plus Erpenbeck’s ‘I3-M11’ extension) and the ribosomal fragment (18S-ITS1-5.8S-ITS2-28S) showed no genetic differences (100% similarity according to Kimura p distances) with the previous samples (SPO8, SPO16, SPO20) classified as *Callyspongia (Cladochalina) diffusa*. Please, see previous species above for further details and remarks.

*Callyspongia (Toxochalina)* cf. *pseudotoxa* Muricy & Ribeiro, 1999

Fig 18

**Fig 18:**
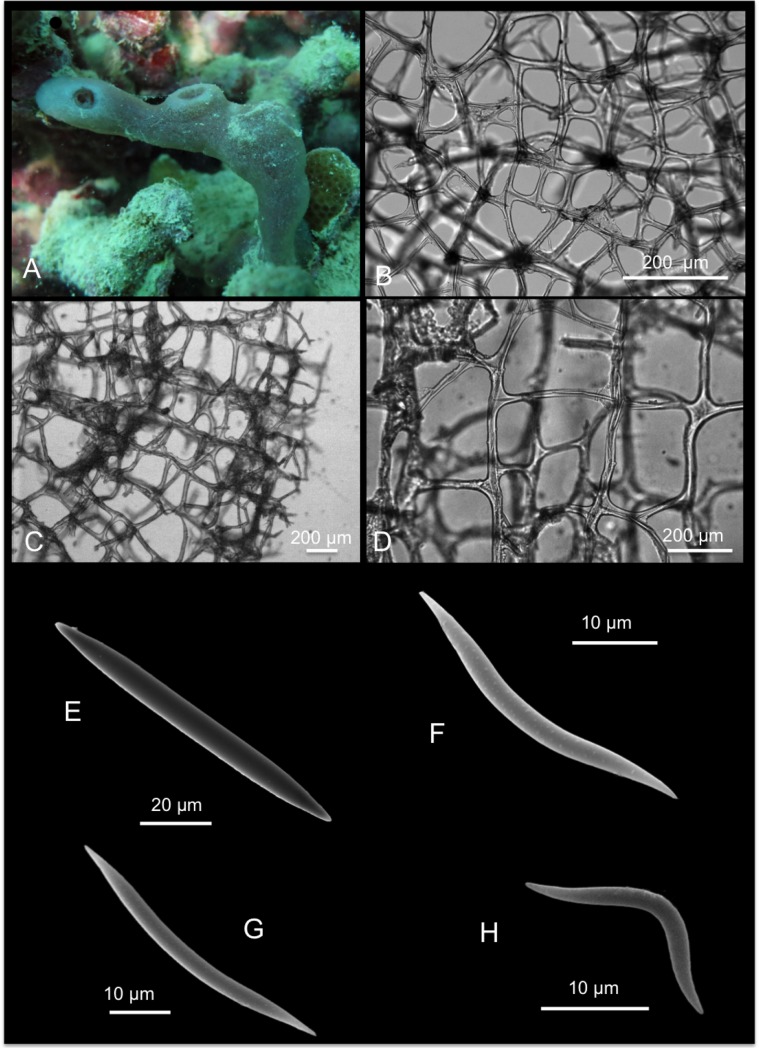
*Callyspongia (Toxochalina)* cf. *pseudotoxa*. A alive specimen; B ectosomal skeleton; C, D choanosomal skeleton; E stout oxea; F, G oxhorn toxas I with variable angle of curvature; H oxhorn toxa II.

*Callyspongia (Toxochalina)* cf. *pseudotoxa* Muricy & Ribeiro, 1999: 94 [83].

Material: SPO 34, reef 25, 3 m.

Description: Erected sponge (Fig 18A) pinkish in colour, light brown in alcohol. The examined sample consists in a branch about 7 cm long and 1.5 cm thick. Slightly elevated large oscules up to 7 mm are evident in the preserved specimen; surface is optically smooth. The sponge is fibrous, quite firm, but resistant to tear.

Skeleton: The ectosomal skeleton (Fig 18B) is a regular, tangential web of quadrangular or polygonal meshes (about 40–570 μm wide), delimitated by unispicular primary and secondary fibres (about 10–45 μm in diameter). The choanosome (Fig 18C and 18D) is a regular network of rectangular mesh (200–400 μm large) formed by paucispicular ascending fibres (25–60 μm in diameter) connected at right angle by secondary unispicular fibres (20–25 μm).

Spicules: Stout oxeas (Fig 18E), in general straight, with acerate tips 95-(101.6±3.6)-105 x 5-(7±0.7)-7.5 μm. Oxhorn toxas in two size categories: I with variable angle of curvature so that the shape may vary from a typical toxa to an oxea, slightly bent in the middle 35-(44.4 ± 4.4)- 2.5–3.7 μm (Fig 18F and 18G); II small, quite uncommon, in general with a more regular shape 22-(26±4.1)-30 x 2.5 μm (Fig 18H).

Distribution: *C*. *pseudotoxa* was recorded in Brazil only (Arraial do Cabo, Rio de Janeiro).

Remarks and discussion: The present specimen is morphologically indistinguishable from the Brazilian one. They share the same colour (pinkish alive and cream in alcohol), skeletal organization and comparable size of the meshes and of the diameter of the fibres; the species described by Muricy & Ribeiro [83] has unispicular fibres in the ectosome, 15–70 μm in diameter, rectangular/ovoid meshes 120–560 μm wide, in the choanosome primary paucispicular fibres 20–70 μm in diameter and secondary, unispicular fibres 10–60 μm in diameter, create meshes of about 125–625 μm wide; the spicule feature is the same with oxeas 89–122 x 1.6 10 μm, oxhorn toxas I 19.6–40.7 x 2–4 μm and II 31.8 x 2–4 μm. In particular *C*. *pseudotoxa* is strongly characterized by the possession of oxhorn toxas “with peculiar shape […] reminding an oxea bent in the middle” that give the name to the species [83]; this peculiar kind of spicule is also present in the Hawai’ian specimen. This represents the first record of this species for Hawai’i.

This study reports the first barcoding data on this sponge species. The COI marker revealed no species separation with respect to a *C*. *fallax* from Florida, USA (100% match) and with a clone of *C*. *ramosa* from New Zealand (99.4% similarity). The divergence with *C*. *siphonella* and *Callyspongia* sp. was 3.8% and 4.8% respectively, whereas p distances calculated against our *C*. *diffusa* sequences and with a reference from *C*. *plicifera* ranged 7.3% to 8.3%. This suggests that the COI marker might not be able to discriminate some species within *Callyspongia*. As mentioned in the previous sponge description, the ITS ribosomal sequence recorded 13.1% divergence with all *C*. *diffusa* from our dataset, and with a *Callyspongia* sp. reference.

Family Chalinidae Gray, 1867

*Cladocroce burapha* Putchakarn, de Weerdt, Sonchaeng & Van Soest, 2004

Fig 19

**Fig 19:**
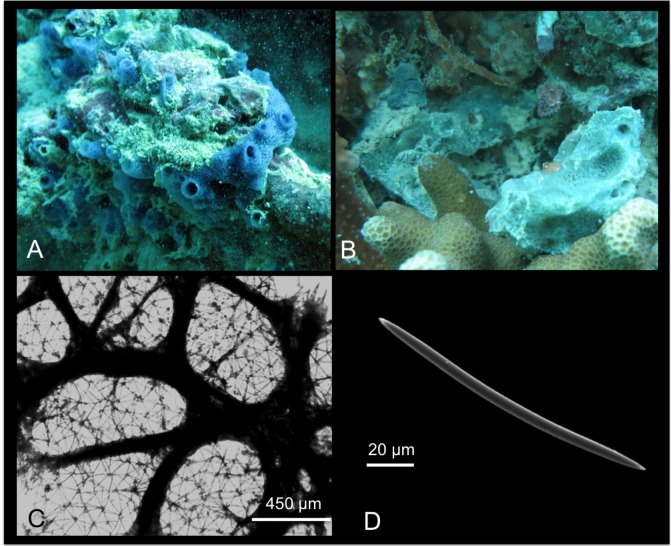
*Cladocroce burapha* Putchakarn, de Weerdt, Sonchaeng & Van Soest. A specimen SPO 29 *in situ*; B specimen SPO 32 *in situ*; C ectosomal skeleton; D oxea.

*Cladocroce burapha* Putchakarn, de Weerdt, Sonchaeng & Van Soest, 2004: 113 [84].

Material: SPO 29, reef 20, 5 m; SPO 32, reef 22, 3 m.

Description: The specimen SPO 29 (Fig 19A) consists of short, partially fused tubes opening in an apical osculum. The colour of this specimen is light blue; SPO 32 is a massive and irregular sponge growing between coral branches (Fig 19B); the color of this specimen is light grey. The preserved specimens turn light cream in ethanol. In both cases, the surface is smooth, but the ectosomal web is clearly visible in the living specimens. Texture is soft and compressible.

Skeleton: The ectosomal skeleton is a regular, isotropic, unispicular web (Fig 19C); the choanosomal skeleton is an unispicular web similar to the ectosomal one, enforced by plurispicular tracts, 60–300 μm in diameter, creating circular or ovoid meshes 200–980 μm wide.

Spicules: Oxeas (Fig 19D), straight or slightly curved with acerate tips. Measurements are shown in Table 12.

**Table 12:** Spicule measurements of *C*. *burapha*.

	Oxeas (μm)
SPO 29	130-(147±10)-167 x 5-(6.5±0.9)-8.7
SPO 32	125-(138.2±10.1)-157.5 x 3.7-(6.2±1.5)-8.7

Distribution: This species was described for Thailand and was recorded in Indonesia [12].

Remarks and discussion: Putchakarn et al. [84] put in evidence the high morphological variability of this species in the colour and spicule size. The Hawai’ian specimens fit with the species paratype described for Thailand in the general morphology, and also in the light blue colour and the larger size of the oxeas (141–166.8–171 x 6–7.5 μm). The Hawai’ian specimens though, registered larger diameter in the choanosomal fibres.

This is the first record of *C*. *burapha* for Hawai’i. We provide the ribosomal partial 18S-28S marker and also the standard Folmer COI partition (without the extension). These are the only existing sequencing data on this sponge species. The COI sequence from SPO29 and SPO32 showed 99.9% similarity, and since no congeneric nucleotide entries are available on GenBank, no relevant species demilitation validation could be estimated. Some sponges within the genus *Haliclona* (e.g., *H*. *implexiformis*, *H*. *oculata*, *H*. *xena*) showed low genetic p distances (>2.5%), however this genus seems to be genetically quite heterogeneous.

*Haliclona (Soestella) caerulea* (Hechtel, 1965)

Fig 20

**Fig 20:**
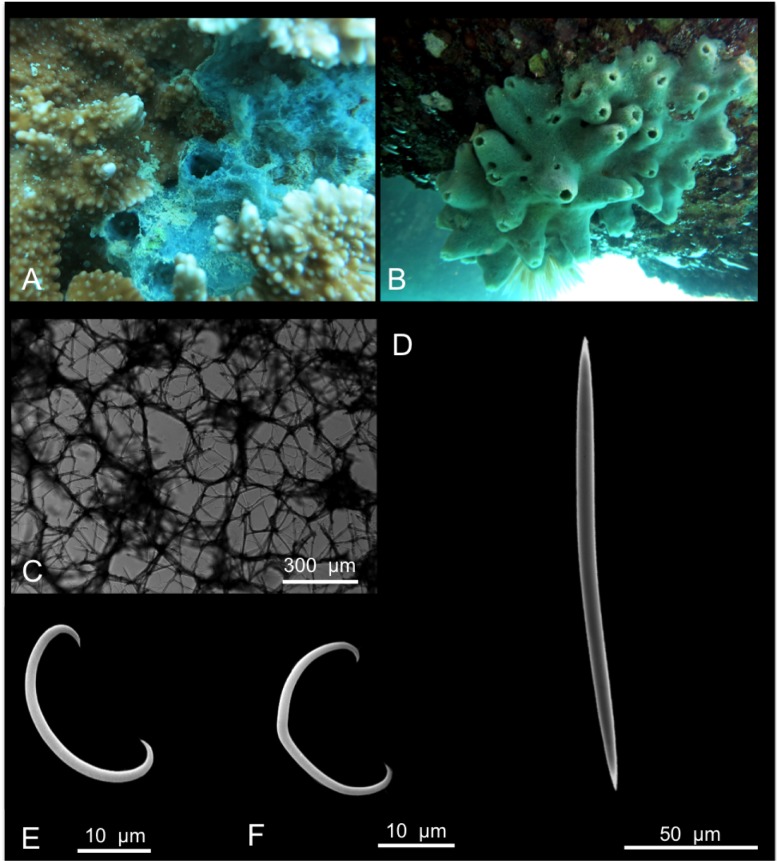
Haliclona (Soestella) caerulea. A specimen SPO31 *in situ*; B specimen SPO23 *in situ;* C ectosomal skeleton; D oxea; E sigma; F centrangulated sigma.

*Sigmadocia caerulea* Hechtel, 1965: 30 [85]; De Weerdt, 2000: 29 [86].

Material: SPO 21, Coconut Is., Lagoon Floating Deck, 0.1 m; SPO 23, Coconut Is., Lagoon Floating Deck, 0.1 m; SPO 31, Reef 22, 3m.

Description: The sponges were irregularly massive, and were found growing among coral branches (SPO 21 and 31, Fig 20A). In some cases specimens can develop long chimneys opening with an apical osculum (Fig 20B, SPO 23). Specimens SPO 21 and SPO 23 were light blue, while SPO 31 was vivid blue; beige when preserved. The surface is smooth, and the texture is soft but elastic.

Skeleton: In the ectosome (Fig 20C) oxeas are organized in a regular unispicular, isotropic reticulation with abundant dispersed sigmas, and form irregular rounded meshes. In the choanosome (Fig 20C) plurispicular tracts of oxeas, creating rounded meshes, are evident.

Spicules: Oxeas (Fig 20D) slightly curved and with sharp ends; sigmas (Fig 20E) C-shaped, often centrangulated (Fig 20F). Measurements are shown in Table 13.

**Table 13:** Spicule measurements of *Haliclona (Soestella) caerulea*.

	Oxeas (μm)	Sigmas (μm)
SPO 21	147-(184.5±14.99–207.5 x 3.7-(5.6±1.3)-7.5	20-(22.5±2.1)-25
SPO 23	152.5-(200.7±16.4)-220 x 7.5-(8.9±1)-10	17.5-(22.5±2.8)-25
SPO 31	150-(174.2±10.7)-187.7 x 3.7-(6.3±1.4)-8.7	15-(20.7±2.3)-22.5

Distribution: Jamaica [85], Puert Rico, Curaçao [87], Virgin Islands, Martinique, St. Vincent, Grenada, Bonaire, Venezuela, Colombia, Belize; Pacific Coast of Panama ([88,86]; Hawai’i and Guam [3].

Remarks and discussion: Hechtel [85] firstly described *H*. *(S*.*) caerulea* in Jamaica on pilings, mangrove roots, and sandy turtle grass beds. Van Soest [87] reports the species from mangrove roots and intertidal rocks in the Caribbean, whereas Wulff [88] noted that eastern Pacific specimens were found on the bases of branching pocilloporid corals. In Hawai’i *H*. (*S*.*) caerulea* has been reported in O’ahu–Pearl Harbour, Honolulu Harbour, Keehi Lagoon, Kewalo Basin, Ala Wai Harbour, and Kane’ohe Bay; Kauai–Nawiliwili Harbour; and Midway Atoll–main harbour, where it is mainly restricted to shallow-water fouling communities (i.e. pier pilings, floating docks) or associated disturbed habitats (i.e. dredged channels and artificial lagoons). It is also found on the roots of the nonindigenous Red Mangrove, *Rhizophora* mangle, native to Florida, West Indies, and South America, which is abundant in Pearl Harbour and Keehi Lagoon. In Kane’ohe Bay, *H*. *(S*.*) caerulea* was described on southeast corner patch reefs as well as on Coconut Island floating docks. *Haliclona (S*.*) caerulea* was not listed in the inventories from de Laubenfels [4,6,8] and Bergquist [9] conducted around Coconut Island (Kane’ohe Bay), where it is now conspicuously abundant. It is improbable that this species had been overlooked during these studies. Therefore, it is considered a recently introduced species also due to its disjunct geographic distribution (Caribbean and Hawai’ian Islands) [5,75]. There are several barcoding records of this species in NCBI database for the COI and the 18S and 28S genes. This study affords two COI standard partitions and one 18S-28S fragment to the public database. The COI sequences from SPO21 and SPO23 were 99.4% equal, and had 99.7% similarity with reference records of conspecifics from Hawai’i, Palmyra and Caribbean. These data confirm the alien origin of this species, and its current cosmopolitan colonization range. The closer congeneric reference was *H*. *simulans* with 4.5% divergence, whereas as other *Haliclona* records diverged >10%. The ribosomal 18S-28S partial markers showed 100% match between SPO21 and SPO23, but these were very different from other congeneric records, displaying from 8% up to 72% p distances.

*Haliclona (Reniera)* cf. *aquaeductus* (Schmidt, 1862) *sensu* de Laubenfels, 1951

Fig 21

**Fig 21:**
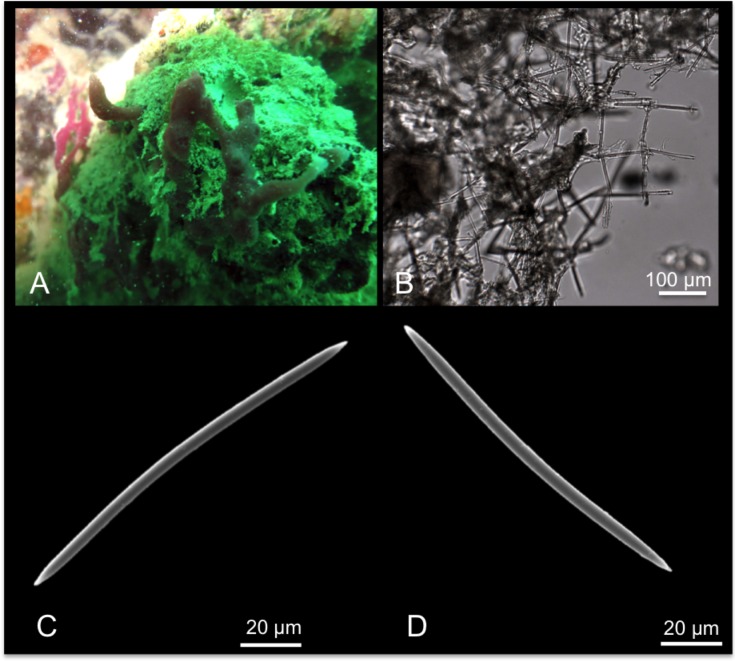
*Haliclona (Reniera)* cf. *aquaeductus sensu* de Laubenfels. A alive specimen; B ectosomal skeleton; C, D oxeas.

*Reniera aquaeductus* Schmidt, 1862: 72 [89].

Material: SPO40, reef 25, 10 m.

Description: Erect sponge consisting of short branched tubes (Fig 21A); the sponge is purple *in situ*, cream when preserved. The consistence is soft and fragile.

Skeleton: Delicate ectosomal, isodictyal skeleton harbouring triangular or quadrangular meshes (Fig 21B); the choanosomal skeleton has the same structure.

Spicules: Oxeas, in general straight or slightly curved (Fig 21C and 21D); they measure 100-(113±5.8)-117.5 x 2.5-(4.5±1)-5 μm.

Distribution: This species was described by Schimdt [89] from the Adriatic Sea. De Laubenfels [6] reported it along the costs of the Island of Hawai’i.

Remarks and discussion: From a morphological point of view our sample fits with the description of the species made by de Laubenfels [6] and by other authors, e.g Griessinger [90].

However, this species belongs to a group of difficult taxonomic determination because of the scarcity, variability and simplicity of the characters [86]. It is very close to the species described by de Laubenfels in 1951 for the Hawai’i Island and determined as *Haliclona (Reniera) aquaeductus* [89]; they share the same external organisation, colour, shape and size of the spicules. This specimen has a cryptogenic origin and, considering the simplicity of its morphological characters and the paucity of the available material, we attribute it to the species *Reniera aquaeductus* Schmidt, 1862 *sensu* de Laubenfels, 1951, pending further studies. Here we provide the first COI (including the extension) and ribosomal sequences for this species. COI sequences revealed congruent p distance values for species delimitation when contrasting with the available congeneric reference records, with dissimilarity values ranging 3–4.3% (with e.g., *H*. *implexiformis*, *H*. *oculata*, *H*. *xena*, *H*. *toxius*, *H*. *cinerea*, *H*. *tubifera*) up to 14% (with *H*. *caerulea*). The ITS region was between 8% and 14% divergent from reference congeneric entries (e.g., *H*. *amboinensis*, *H amphioxi* or *H*. *fascigera*, and in accordance with COI, remarkably dissimilar (up to 66.2%) with respect to SPO21 and SPO23 (*H*. *caerulea*)

Family Niphatidae Van Soest, 1980

*Gelliodes wilsoni* Carballo, Aquilar-Camacho, Knapp & Bell, 2013

Fig 22

**Fig 22:**
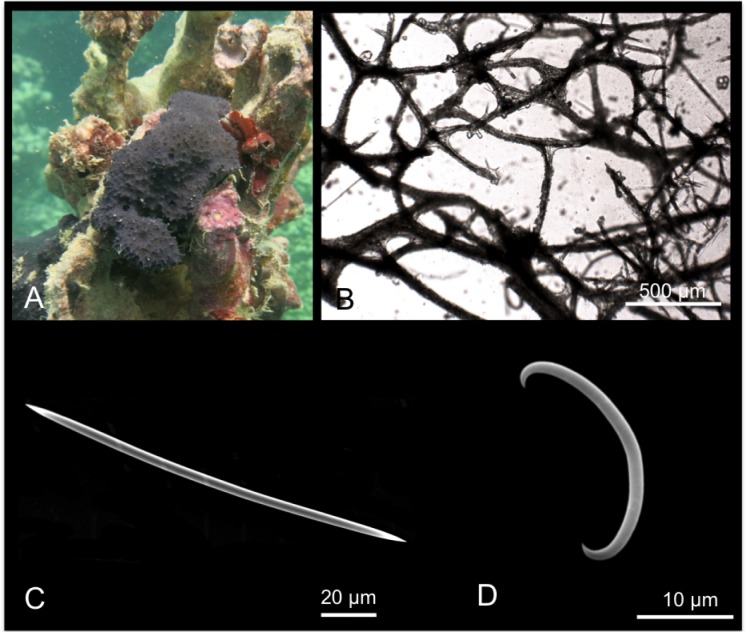
Gelliodes wilsoni. A alive specimen; B choanosomal skeleton; C oxea; D sigma.

*Gellius varius var*. *fibrosa* Wilson, 1925: 388 [91]; Carballo, Aquilar-Camacho, Knapp & Bell, 2013: 770 [92].

Material: SPO10, Coconut Is., Point Lab., 9 m; SPO28, Reef 20, 5m.

Description: Massive or massively encrusting sponge; the colour is dark purple, grey (Fig 22A); dark brownish when preserved; oscules slightly prominent and evident; the surface is spiny with short conules; the texture is relatively elastic and spongy, difficult to tear apart.

Skeleton: In the ectosome and choanosome plurispicular tracts of oxeas about 40–130 μm in diameter create an irregular reticulum with wide meshes up to 600 μm wide (Fig 22B).

Spicules: Oxeas, straight or slightly curved (Fig 21C); sigmas regular in shape (Fig 22D). Measurements are found in Table 14.

**Table 14:** Spicule measurements of *Gelliodes wilsoni*.

	Oxeas (μm)	Sigmas (μm)
SPO 10	142.5-(155.6±7.2)-167 x 3.7-(5±1.5)-7.5	17.5-(17.85±0.9)-20
SPO 28	112-(142.8±10.2)-152.5 x 3-(5±0.9)-6.2	12.5-(15.1±2.4)-20

Distribution: Widely diffused in the Pacific Ocean [92].

Remarks and discussion: *G*. *wilsoni* originates from the Philippines, first reported by Wilson [91] and later by de Laubenfels [93]. But, it is believed that in 1992 it arrived to the Hawai’ian Islands and became abundant since 1997 in O’ahu (leeward coast harbours, and Kane’ohe Bay), Maui (Kahului Harbour) and Kauai (Nawiliwili Harbour). In Guam the introduction is dated in 1999 [5,75].

In the Hawai’ian Islands, *G*. *wilsoni* (= *G*. *fibrosa*) is mainly restricted to shallow-water fouling communities (i.e. pier pilings, floating docks) of the major harbours or associated disturbed habitats (i.e. dredged channels and artificial lagoons) on O’ahu, Kauai and Maui. In Kane’ohe Bay, *G*. *wilsoni* is found on patchy reefs in southeast corner, typically encrusting the shaded underside of plate corals, as well as on Coconut Island floating docks. This conspicuous species is considered nonindigenous, as the sponge experts de Laubenfels [4,6,8] and Bergquist [9] did not mention it in their surveys. The specific classification of the specimens from Hawai’i was recently confirmed by Carballo et al. [92], who collected this species in Kane’ohe Bay and renamed it (emended an homonomy) as *G*. *wilsoni*.

Carballo et al. [92] showed the high phenotypic plasticity of this species in particular in the spicule size and in the sponge shape; the spicule size of our specimen is in the size range recorded by the authors for the Hawai’ian specimens (oxeas 125-(160±12)-188 x 2-(3.8±1.37)-7.5; sigmas 10-(16.6±2.9)-25 μm). Our specimens show wider size for skeletal meshes and fibres. This study reports the first barcoding data for COI and ribosomal genes. Since there were no reference nucleotide entries from conspecific or congeneric representatives, the estimation of species delimitation was inaccessible. Specimens SPO10 ans SPO28 had 100% matching COI sequences, and these were 0.2% divergent from that from *Gelloides* sp. SPO24. In the ribosomal fragment, the sequences from both sponges diverged 0.3–0.8%.

Family Niphatidae sp.

*Gelliodes* sp.

Fig 2D

Material: SPO24, Reef HIMB (Lagoon Floating Deck), 0.1 m.

Description: Dense lobular shaped sponge, opaque dark, grey in colour, with numerous, circular oscules of diverse size visually distinguishable. The surface is optically smooth, and the texture is spongy but elastic, especially in preserved specimens.

Skeleton: In the ectosome, there are polygonal meshes made by plurispicular fibres of oxeas about 50–150 μm in thickness; at the nodes of the meshes groups of oxeas protrude. The choanosome is quite regular, made of plurispicular tracts of oxeas running towards the sponge surface, and connected by transversal paucispicular tracts of oxeas.

Spicules: Oxeas, straight or slightly curved often modified in styles or strongyles (about 160–180 x 5 μm; sigmas quite numerous about 15–20 μm.

Remarks and discussion: We were not successful in retrieving the ribosomal marker amplified for this sample. The COI marker instead matched 99.8% with a *Gelloides wilsoni* SPO10 and SPO28 from this study. From a morphological point of view, *Gelliodes* sp. is different from SPO 10 and SPO 28 in having smooth surface and longer oxeas with modified tips. Since SPO24 consists in a single and small sample and the simplicity of its morphological characters (as already pointed for other Haplosclerid sponges) we prefer to determine this species as a separate taxonomic entity and identified it at genus level only.

Subclass Keratosa

Order Dictyoceratida

Family Dysideidae Gray, 1867

*Dysidea* cf. *arenaria* Bergquist, 1965

Fig 23

**Fig 23:**
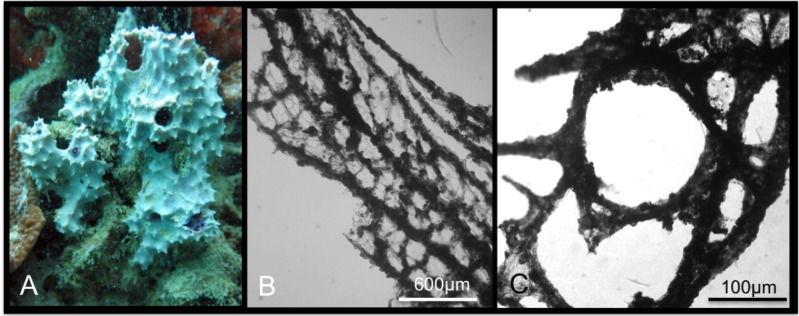
*Dysidea* cf. *arenaria*. A alive specimen; B foreign sand grains and spicules on sponge surface organized in parallel tracts; C fibres not distinct in primary and secondary.

*Dysidea arenaria* Bergquist, 1965: 144 [71].

Material: SPO 39, reef 1, 4.7 m.

Description: Massive sponge with a strongly conulose surface (Fig 23A). Among the conules, traces of the cored fibres are clearly visible both in the living and preserved sample. Conules are large and slightly elevated. Whitish, grey *in situ*, dark brown when preserved; in alcohol the sponge releases a dark pinkish pigment. The consistence is elastic, but it breaks easily.

Skeleton: On the sponge surface, scattered foreign sand grains and spicules could be distinguished, often organized in parallel tracts (Fig 23B) (about 50–140 μm in diameter) creating a regular web, visible at the naked-eye. In the internal parts, fibres (40–150 μm long) not distinguishable as primary or secondary are disposed completely cored with foreign detritus and create a fairly regular web (Fig 23C).

Distribution: Only known from its type locality (Palau, Western Caroline); more recently it was recorded in Hawai’i, first in Pearl Harbour in 1996, and later it was reported by Coles et al. [75] as cryptogenic.

Remarks and discussion: The species is characterized by the lack of distinct primary and secondary fibres and by the presence of a sand cortex, 85–100 μm thick [71]. The specimen collected by us is close to the species described by this author, but it lacks a thick sand cortex as only sand grains, scattered and organized in tracts, are present on the surface. Barcoding data is available in NCBI database for COI and ribosomal genes. We submitted the partial 18S-28S fragment of our voucher, which yielded distances ranging 17.9–23.8% (with *D*. *arenaria*, *D*. cf. *herbacea*, *D*. cf. *pallescens*) and up to 70.5% (*D*. cf. *granulosa*) against reference conspecifics. Amplification did not succeed for COI partitions.

*Dysidea* sp. 1

Fig 2E and 2F

Material: SPO12, Reef HIMB, 9 m. SPO 18, Reef HIMB (Lagoon Floating Deck), 0.3 m.

Description: SPO 12 is a light purple, translucent specimen, with shimmering fibres. The sponge has a conulose surface and is very cushiony to touch. SPO 18 is a light cream very translucent sponge, highly thorny in shape, with conspicuous oscules and shimmering fibres, and is very cushiony to touch. The sponge turns dark beige in alcohol.

Skeleton: Not distinguishable primary and secondary fibres about 50–200 μm in diameter. Specimens are rich in sand grain and foreign spicules. Spongin is visible and in some sections, small fibres are free from coring.

Remarks and discussion: It was impossible to amplify the Erpenbeck’s ‘I3-M11’ extension of the COI marker region. The standard 5’ Folmer partition from both specimens (SPO 12 and SPO 18) resulted ~99.8% similar to each other, and were 2.7% divergent from SPO17, suggesting enough genetic species delimitation between *Dysidea* sp. 1 and *Dysidea* sp. 2. The p distance values against available reference conspecific sequences (*D*. *arenaria*, *D*. *avara*, *D*. *fromdosa*) ranged 5.7% to 7.2% for the three sponge specimens. The ribosomal fragments from both *Dysidea* sp. 1 (sponges SPO12 and SPO18) were 100% matching with each other and also with three references of *D*. *arenaria* from New Caledonia. Instead, SPO 17 displayed a 3.3% divergence, supporting again the existence of species delimitation between *Dysidea* sp. 1 and *Dysidea* sp. 2. The other available conspecific records were over 10% dissimilar (*D*. *arenaria*, *D*. cf. *herbacea*, *D*. cf. *pallescens*, and *D*. cf. *granulosa*).

*Dysidea* sp. 2

Fig 2G

Material: SPO17, Reef HIMB (Lagoon Floating Deck), 0.3 m.

Description: Cerulean blue sponge, massive, evident conules, very soft to touch.

Skeleton: Fibres cored with sands grain and spicules; they are about 50–150 μm in diameter.

Remarks and discussion: The very small size of the specimen prevented the specific determination. The barcoding data submitted for this specimen included the standard 5’ Folmer partition for the COI marker (not the Erpenbeck’s ‘I3-M11’ extension), as well as the ribosomal 18S-28S partial fragment. Both markers revealed >2.5% divergence to genetically separate *Dysidea* sp. 1 from *Dysidea* sp. 2. Please, see above species description of *Disidea* sp. 1 for details).

Subclass Verongimorpha

Order Chondrillida

Family Chondrillidae Gray, 1872

*Chondrilla mixta* Schulze, 1877

Fig 24

**Fig 24:**
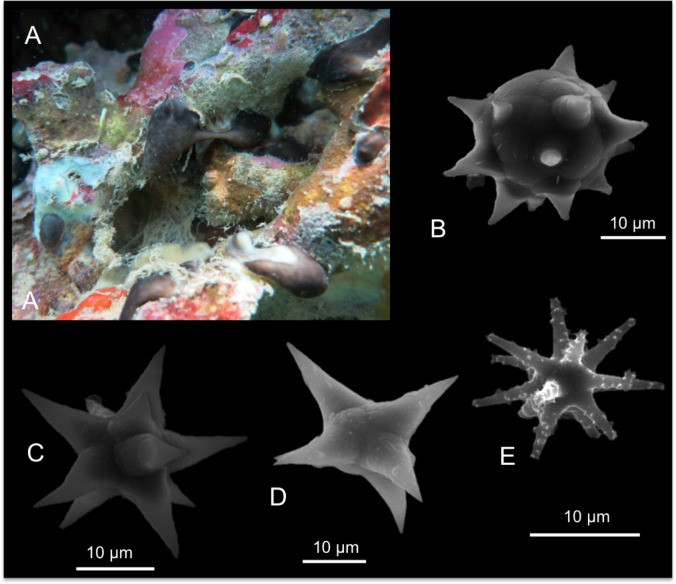
Chondrilla mixta. A alive specimen; B-D sphaerasters; E oxysphaeraster.

*Chondrilla mixta* Schulze, 1877: 116 [94]; *Chondrilla nuda* Lendenfeld, 1987: 105 [79].

Material: SPO11, Coconut Is., Point Lab., 9 m.

Description: Thickly encrusting sponge forming small lobes (Fig 24A). The colour in living specimen is dark brown, or dark grey with lighter spotted areas; the sponge turns black when preserved. The surface is smooth and shiny; the texture is firm and compact.

Skeleton: Rounded sphaerasters are dispersed meanly in the ectosome. In the internal part oxysphaerasters are scattered in the tissue.

Spicules: The sphaerasters have a large centrum and smooth rays (Fig 24A–24D); numerous sphaeraster with shorter rays that may be also reduced in number; in this way sphaerasters with very few rays or completely without rays are numerous; they measure 12.5-(29.4±6)-40.5 μm in diameter. The oxysphaerasters (Fig 24E) have a smaller centrum and fewer conical and spined rays, often slightly bent; they are slightly smaller than the other category and are about 12–20 μm in diameter.

Distribution: East African; Indian Ocean, Red Sea, Indonesia.

Remarks and discussion: The Hawai’ian specimen fits with the species *C*. *mixta*, in the general external morphology, sphaerasters disposition in the skeleton, spicule shape and size.

This species is characterized by its lobular organisation, colour and shape remanding of *Chondrosia reniformis*. Two kinds of sphaerasters are also not very diffused among *Chondrilla* species. This is a new record for Hawai’i. Our sequences for COI and ribosomal partial genes are the first barcoding data submitted for this species. The COI sequence displayed over 99.2% similarity with all the available conspecific *Chondrilla* records (*C*. aff. *nucula*, *C*. *caribensis*, *C*. *australiensis*) coming from very distant locations around the planet. In accordance to this finding, other authors had already noticed that different *Chondrilla* sponges that can be identified through allozymes, ribosomal sequences and even conventional taxonomy, cannot be separated as diverse species if applied a 2.5% divergence threshold with the COI marker [15]. The ribosomal ITS fragments by contrast gave us robust resolution, and our specimen diverged from other conspecifics, such as *C*. *australiensis*, *C*. *nucula* and an Australian *Chondrilla* sp. by 9.4%, 11.9% and 11.8% p distance respectively.

Order Verongiida

Family Pseudoceratinidae

Genus *Pseudoceratina*

*Pseudoceratina purpurea* (Carter, 1880)

Fig 25

**Fig 25:**
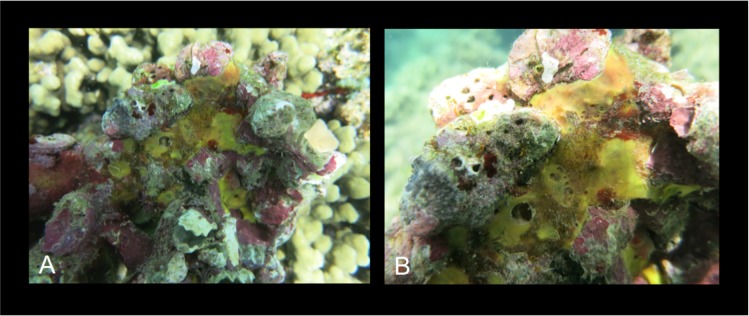
Pseudoceratina purpurea. A live specimen; B magnification with in evidence the smooth surface.

*Aplysina purpurea* Carter, 1880: 36 [95]; *Hexadella pleochromata* de Laubenfels, 1950: 10 [4].

Material: SPO9, Reef HIMB (Point Lab), 5 m.

Description: Yellow, dense very opaque, even sponge with circular wide oscules openings (Fig 25A and 25B). Specimens grow very thin in crevices and under rocks and rubble, and profiting holes and cracks. The living material turns dark purple to black when cut, and is smooth to touch.

Skeleton: Thin, sparse and rare, dendritic and laminated fibres, about 40–50 μm in diameter.

Distribution: Widely distributed in the Indo-Pacific and also present in Hawai’i.

Remarks and discussion: The small dimension of the sample put difficulties for the identification. De Laubenfels in 1950 [4] described the species *Hexadella pleochromata* as encrusting, sulphur-yellow coloured, with smooth surface. He defined the “endosome completely askeletal” thus confirming the scanty presence of fibers and their weakly development as reported also by Carter [95]. This species was later considered synonymous of *P*. *purpurea* Carter by Bergquist [9], who examined de Laubenfels’ holotype.

We present the first barcoding data for this sponge species, which includes the standard 5’ Folmer partition and the Erpenbeck’s ‘I3-M11’ extension of COI, and the ribosomal 18S-ITS1-5.8S-ITS2-28S fragment. Similar to what we reported in *Chondrilla*, the COI marker again was not able to separate species and displayed over 99.5% similarity with other the available geographically distant conspecific records (*P*. *arabica*, *P*. *durissima*, *P*. *verrucosa* and several *Pseudoceratina sp*.). There were no conspecific or congeneric ITS ribosomal sequences to support our species delimitations and further support the morphological classification for this marker. A shorter curated fragment of 273bp covering partially the 18S-ITS1-5.8S-ITS2-28S fragment from several *P*. *arabica* clones from Palau revealed 2.6% dissimilarity with our *P*. *purpurea* SPO9.

## Discussion & remarks

In the current study we examined 30 species of Porifera along Kane’ohe Bay, of which 24 are described and discussed in detail. Barcoding data for the COI marker and the fragment covering the ribosomal genes and spacers 18S, ITS-1, 5.8S, ITS-2 and 28S were submitted to GenBank NCBI public database for the sponges examined (60 nucleotide sequences newly submitted associated to this study). Most of these DNA sequences are the first sequencing records for the corresponding species (88.3%), and will serve as references for future research.

To the best of our knowledge, at least six of the sponges analysed here have not been previously reported in the area [6,9,10,96] and represent new records for Hawai’i: *Hymeniacidon gracilis*, *Cliona dissimilis*, *Callyspongia (T*.*) pseudotoxa*, *Cladocroce burapha*, *Chondrilla mixta* and *Monanchora clathrata*. In total, 141 sponges (130 published and 11 unpublished records; S1 Table) had previously been cited in Hawai’ian regions; of these, only seven are believed to be indigenous, whereas most of the new entries are likely NIS (non–indigenous species) that have been introduced in the past years or considered cryptogenic [10,11,75, 27,12]. We suspect that future surveys will bring about new records, probably as a result of constant faunal introductions to O’ahu. The isolated location of the Hawai’ian Archipelago along with its young geological age [97] contribute to the relatively low biodiversity of the area, in comparison to other biogeographic sectors of the Western and Central Pacific. These factors also promote high levels of endemism (about 20% of marine invertebrates), but most of the endemic species are in the actuality very poorly represented [98,99]. It is hypothesised that NIS have displaced a considerable number of endemic species [1]. In 1999 the number of aquatic alien invertebrates was documented to be 287, about 7% [3,5]. An event that triggered substantial species inoculation in O’ahu was the relocation of the floating drydock Machinist from Subic Bay, Philippines in 1992 [78]. This fact favoured introductions from the Indo-West Pacific, but indirectly also from other farther locations [75,3].

Sponges have low dispersal capabilities, in part due to their lecitotrophic larval strategies, thus it is considered unlikely that there has been a great deal of alien colonization without artificial drivers [28]. The most probable mechanism of introduction of Porifera is unintentional, as part of the fouling communities on ships’ hull [75]. For this reason, harbours and areas where boats and vessels commonly dock are the most infested with allochthonous species [3]. The sponge fauna reported from Hawai’i, similarly to the other invertebrates, consists of a small group of endemic species, and many species that distribute throughout the Western Pacific, Red Sea, Australia, and even the Caribbean (57 endemic species and 7 recognized as introduced; S3 Table). Indeed, sponge surveys are constantly reporting new records of introduced or cryptogenic species, which are often morphologically indistinctive from Atlantic and Caribbean morphotypes [12]. Our results follow this trend: One of the species (*C*. *(T*.*)* cf. *pseudotoxa*) was only known from Brazil and could represent a new introduction, six species were new records for the area and have a wide distribution in the Indo-Pacific Ocean; two species were endemic to Hawai’i, and the rest were mainly represented by cryptogenic NIS [75].

Most inoculated sponges in Hawai’i appear to be playing a non-invasive role as spatial competitors [78,5,3]. Unlike these relatively benign species, *Mycale (Mycale) grandis* does seem to represent a real threat as an overgrowing agent on scleractinian corals (i.e., *Porites compressa*, *Montipora capitata*) that further can weaken reef framework structures [74,2]. Actually, the most intense sponge research up to date comes from surveys performed by the Hawai’i Coral Reef Initiative and the Bishop Museum monitoring distribution, abundance, growth rates and competitive impacts of *Mycale (M*.*) grandis* around K-Bay [74,2].

The sponge fauna in Hawai’ian reef systems is rich and diverse and has an urgent need to be characterized [10]. Almost nothing is known about species distribution, abundance and coverage, and the lack of knowledge becomes more acute in functional ecological aspects. As mentioned, a few older studies compile inventories where morphological descriptions are given but these are often vague and/or use obsolete nomenclatures [6,9,10,95]. The most recent reports basically focused on the invasiveness of *Mycale (M*.*) grandis* [74,2]. Often, taxonomic consultation is the limiting factor to develop research, especially since the number of classical taxonomists is in decline. An important constraint in the taxonomy of Porifera is that databases still need to be well populated and cover a good representation of the known diversity. Also some of the available morphological classifications need to be revised for accuracy, and the submitted sequences curated to match the correct morphospecies. In most animal phyla COI barcoding allow to discriminate species, with the majority of groups reporting > 8% congeneric sequence divergence [14]. Nonetheless, in diploblastic metazoans and sponges the standard COI marker partition does not always disclose sufficient variability for species diagnosis, when applying a 2% divergence threshold [14,15,17]., Here five out of the 16 genera suitable for interspecific distance comparisons revealed a < 2% divergence. The additon of adjacent extensions to the standard COI partition are recommended to improve resolution in Porifera and Cnidaria; and at least in some sponge taxa this approach seems to be resolving better than in cnidarians [18,100]. In our data set, the ‘Erpenbeck I3-M11’ extension [18] seemed to boost species discrimination, especially in *Spheciospongia*, *Iotrochota*, *Tedania*, *Lissodendoryx*, *Callyspongia* and *Haliclona*. ABGD method is efficient in species delimitation when intraspecific diversity is lower than interspecific diversity [31], but is less resolutive where this premise is not met. The recursive partitions were the most resolutive and more consistent with our morphospecies respect to the initial partitions, especially for *Callyspongia* and *Haliclona*. Still, with the recursive partition several species in *Chondrilla*, *Pseudoceratina* and *Iotrochota* could not be separated; while for *Monanchora* and *Tedania* some conspecific sequences were detected as divergent species. Besides the ‘Erpenbeck I3-M11’ extension the inclusion of nuclear markers (e.g., 18S-ITS1-5.8S-ITS2-28S fragment) have proved to facilitate identification. For our analyses here we did not find any conspecific or congeneric sponge reference in GenBank for which the three partions (COI 5′- ‘Folmer’ partition, COI ‘Erpenbeck I3-M11’ extension and 18S-ITS1-5.8S-ITS2-28S fragment) were available. Initiatives that link DNA barcoding, providing a mitochondrial and nuclear markers, along with morphological descriptions (e.g., SBD) represent robust tools for species identification [15,27]. Non-experts can approximate target species identification through costless, straightforward molecular procedures by obtaining the sequence of a barcoding marker region and searching for a matching reference in a database, and accompanying photographic material and simple morphological observations. With this catalog, implemented in the SBD and interconnected with on-line barcoding platforms [23,27], we hope to afford a guide for taxonomy consultation to assist sponge studies in the Hawai’ian waters.

## Supporting information

S1 Fig Recursive automatic barcode gap detection on ABDG. Results are based on COI sequences of our sponge dataset plus reference sequences from GenBank.
(EPS)

S1 Table Distance matrix. Values obtained from ABGD method [1] analysis based on Kimura 2-parameter pairwise distances [2] for the COI fragment, including our sponge sequences and selected reference sequences downloaded from GenBank, with their corresponding accession numbers. References are coded 1–2 according to the list provided on the table footnotes.
(XLSX)

S2 Table Estimates of evolutionary divergence between sequences. Analysis was conducted in MEGA6 [2]. Analyses were conducted using the Kimura 2-parameter model [1]. The rate variation among sites was modeled with a gamma distribution (shape parameter = 1). References are coded 1–2 according to the list provided on the table footnotes.
(XLSX)

S3 Table Compilation of sponge records from Hawai'i, with species geographic distribution and supposed origin. Status–C: cryptogenic, E: endemic, I: introduced and WD: Widely distributed in the Indo-Pacific ocean. References are coded 1–13 according to the list provided on the table footnotes.
(XLSX)
